# A Smartphone-Based Intervention With Diaries and Therapist-Feedback to Reduce Catastrophizing and Increase Functioning in Women With Chronic Widespread Pain: Randomized Controlled Trial

**DOI:** 10.2196/jmir.2249

**Published:** 2013-01-07

**Authors:** Ólöf Birna Kristjánsdóttir, Egil A Fors, Erlend Eide, Arnstein Finset, Tonje Lauritzen Stensrud, Sandra van Dulmen, Sigrid Hørven Wigers, Hilde Eide

**Affiliations:** ^1^Institute of NursingFaculty of HealthOslo and Akershus University College of Applied SciencesOsloNorway; ^2^Department of Behavioral Sciences in MedicineFaculty of MedicineUniversity of OsloOsloNorway; ^3^Department of Psychiatry; National Competence Centre for Complex Symptom DisordersSt Olav University HospitalTrondheimNorway; ^4^Department of Public Health and General PracticeFaculty of Medicine, General Practice Research UnitNorwegian University of Science and TechnologyTrondheimNorway; ^5^edesignTønsbergNorway; ^6^NIVEL (Netherlands Institute for Health Services Research)UtrechtNetherlands; ^7^Department of Primary and Community CareRadboud University Nijmegen Medical CentreNijmegenNetherlands; ^8^Faculty of Health SciencesBuskerud University CollegeDrammenNorway; ^9^Jeløy Kurbad Rehabilitation CentreMossNorway

**Keywords:** Widespread Chronic Pain, Fibromyalgia, Self-management, Mobile phones, Internet, Cognitive Therapy, Catastrophization, Recurrence

## Abstract

**Background:**

Internet-based interventions using cognitive behavioral approaches can be effective in promoting self-management of chronic pain conditions. Web-based programs delivered via smartphones are increasingly used to support the self-management of various health disorders, but research on smartphone interventions for persons with chronic pain is limited.

**Objective:**

The aim of this trial was to study the efficacy of a 4-week smartphone-delivered intervention with written diaries and therapist feedback following an inpatient chronic pain rehabilitation program.

**Methods:**

A total of 140 women with chronic widespread pain who participated in a 4-week inpatient rehabilitation program were randomized into 2 groups: with or without a smartphone intervention after the rehabilitation. The smartphone intervention consisted of 1 face-to-face session and 4 weeks of written communication via a smartphone. Participants received 3 smartphone diary entries daily to support their awareness of and reflection on pain-related thoughts, feelings, and activities. The registered diaries were immediately available to a therapist who submitted personalized written feedback daily based on cognitive behavioral principles. Both groups were given access to a noninteractive website after discharge to promote constructive self-management. Outcomes were measured with self-reported questionnaires. The primary outcome measure of catastrophizing was determined using the pain catastrophizing scale (score range 0-52). Secondary outcomes included acceptance of pain, emotional distress, functioning, and symptom levels.

**Results:**

Of the 140 participants, 112 completed the study: 48 in the intervention group and 64 in the control group. Immediately after the intervention period, the intervention group reported less catastrophizing (mean 9.20, SD 5.85) than the control group (mean 15.71, SD 9.11, *P*<.001), yielding a large effect size (Cohen’s *d*=0.87) for study completers. At 5-month follow-up, the between-group effect sizes remained moderate for catastrophizing (Cohen’s *d*=0.74, *P*=.003), acceptance of pain (Cohen’s *d*=0.54, *P*=.02), and functioning and symptom levels (Cohen’s *d*=0.75, *P*=.001).

**Conclusions:**

The results suggest that a smartphone-delivered intervention with diaries and personalized feedback can reduce catastrophizing and prevent increases in functional impairment and symptom levels in women with chronic widespread pain following inpatient rehabilitation.

**Trial Registration:**

Clinicaltrials.gov NCT01236209; http://www.clinicaltrials.gov/ct2/show/NCT01236209 (Archived by WebCite at http://www.webcitation.org/6DUejLpPY)

## Introduction

Chronic widespread pain is a common cause of suffering. An estimated 4% to 10% of the adult population experiences chronic widespread pain, ie, musculoskeletal pain lasting for more than 3 months not caused by an identifiable physical pathology [1-5]. This pain is often accompanied by other symptoms, including fatigue, sleep disturbance, and emotional distress [2]. A subgroup meets the criteria for fibromyalgia syndrome, where in addition to the chronic pain, the pain thresholds are reduced and tenderness in more than 10 of 18 specified trigger points is identified [2,3]. The development and maintenance of chronic widespread pain and fibromyalgia involve a complex dynamic process with biological, cognitive, and psychosocial factors. The cause or underlying mechanisms are still not clearly identified and no single cure is available. Maladaptive thoughts and feelings seem to play an important part in the negative spiral resulting in the maintenance of chronic pain [6]. Multidimensional rehabilitation, including physical exercise and cognitive behavioral therapy (CBT), is recommended as treatment [7-8]. A key element is self-management, eg, balancing activity and rest, stress management, emotion regulation, and doing appropriate physical exercises [6-10]. However, relapse of symptoms is not uncommon [8,11,12] because self-management can be challenging due to the nature of the symptoms. Few studies have examined home-delivered interventions that aim to support self-management of chronic pain following rehabilitation [11-13].

### Pain Conditions and Web-Based Interventions

Internet-based interventions using cognitive behavioral approaches can be effective in promoting self-management of chronic pain conditions [14-16]. Web-based programs delivered through smartphones are increasingly used to support the self-management of various health disorders; however, research on smartphone interventions for patients with chronic pain is limited [17]. Among the advantages of using smartphones rather than the traditional personal computer s are their small size and mobility, making self-management support available to the user in most situations [17]. Diaries with questions intended to support awareness and reflection are made available on the phone and the registered information can be submitted to a website and made instantly available to a therapist. Feedback can be automatically delivered and tailored to the registered information to some extent, or it can be even more personalized by a therapist [18-20]. In a recent study, a panel of health care professionals and people experiencing chronic pain discussed characteristics of a successful Internet self-management program. Important features included assisting patients to be more aware of their patterns of behavior and psychological experience, supporting the pursuit of personal goals and values-based behavior, and by using a small and mobile device for real-time monitoring and response [21]. The number of pain self-management applications for smartphones has increased exponentially since 2009 [22]. In 2010, more than 90 applications offering support in the self-management of chronic pain were available in application stores. There is a need for research in this field because many applications seem to have been developed without the involvement of a health care professional and, to our knowledge, none have been tested in randomized trials [22].

### Theoretical Model

Cognitive and emotional factors influence the pain experience [23]. Among the psychological constructs that can play an important role in the development and maintenance of chronic pain is catastrophizing [6,23,24]. Pain-related catastrophizing includes the tendency to ruminate about and magnify symptoms, to expect the worst, and to feel helpless regarding self-management [25]. Catastrophizing tends to discourage patients from committing to their valued behavior and it has consistently been found to predict distress and disability [6,26,27]. In rehabilitation, catastrophizing is targeted in a number of ways, such as with CBT and exercise programs [6,28]. However, interventions delivered in the patient’s private environment, supporting awareness of maladaptive thoughts and feelings, and providing personalized feedback may further help reduce catastrophizing [13,18]. A mobile phone-delivered intervention with diaries and daily CBT-based feedback has been found to reduce catastrophizing thoughts in patients with irritable bowel syndrome and these effects were maintained at a 3-month follow-up [18].

Acceptance and Commitment Therapy (ACT) is a third-generation CBT based on the notion that suffering may largely be caused by thinking about painful experiences rather than the experiences themselves [29]. Suffering can be reduced through mindfulness, acceptance, and committed action [29]. ACT has been found to be effective for people with various chronic health disorders [30], and has been used successfully to reduce catastrophizing and disability in chronic pain patients [31-33]. The goals are to promote psychological flexibility, such as acceptance of, rather than struggling with, unwanted thoughts, emotions, and symptoms (eg, pain or catastrophizing) and to increase commitment to personal values [28,29,34]. A person’s values are described as his or her desired way of being within various life domains (eg, being a caring friend). Values differ from goals in that they can never be fully obtained, but can give a continuous sense of motivation, direction, and purpose [28]. The focus on values is also evident in the self-determination theory (SDT) that states the importance of perceiving behavior as self-determined for intrinsic motivation to be maintained [35]. According to the SDT, context-specific feedback can play a role in enhancing intrinsic motivation to maintain behavior [35]. Guidance was also found in the elaboration likelihood model of persuasion theory [36]. This theory specifies how information can be constructed and presented to enhance either cognitive elaboration or emotional elaboration intending to influence behavior change. Elements focused on in this study are repetition, personal relevance and involvement influencing the cognitive level, and influencing emotional pathways through emotion recognition, mindfulness exercises, and empathic communication.

### Aims of the Study

We hypothesized that receiving personalized feedback shortly after having registered pain-related thoughts, feelings, and self-management activities in an everyday setting might reduce catastrophizing and increase functioning. The results of our pretrial study of a similar smartphone intervention indicated feasibility and user-friendliness for patients with chronic widespread pain [20].

The present randomized controlled trial investigates the efficacy of a smartphone intervention on catastrophizing, acceptance, emotional distress, values-based behavior, and functioning and symptom level in women with chronic widespread pain who had completed a 4-week inpatient rehabilitation program. For the first 4 weeks after discharge, the intervention group received a Web-based intervention comprising registration of symptoms, thoughts, feelings, and self-management behavior through daily smartphone diaries and written personalized CBT-based feedback. It was hypothesized that the intervention group would show less catastrophizing and emotional distress, more acceptance of pain, and success in values-based living, and improved functioning and symptom levels after completing the intervention period and at a 5-month follow-up compared to a control group.

## Methods

### Study Design

**Figure 1:**
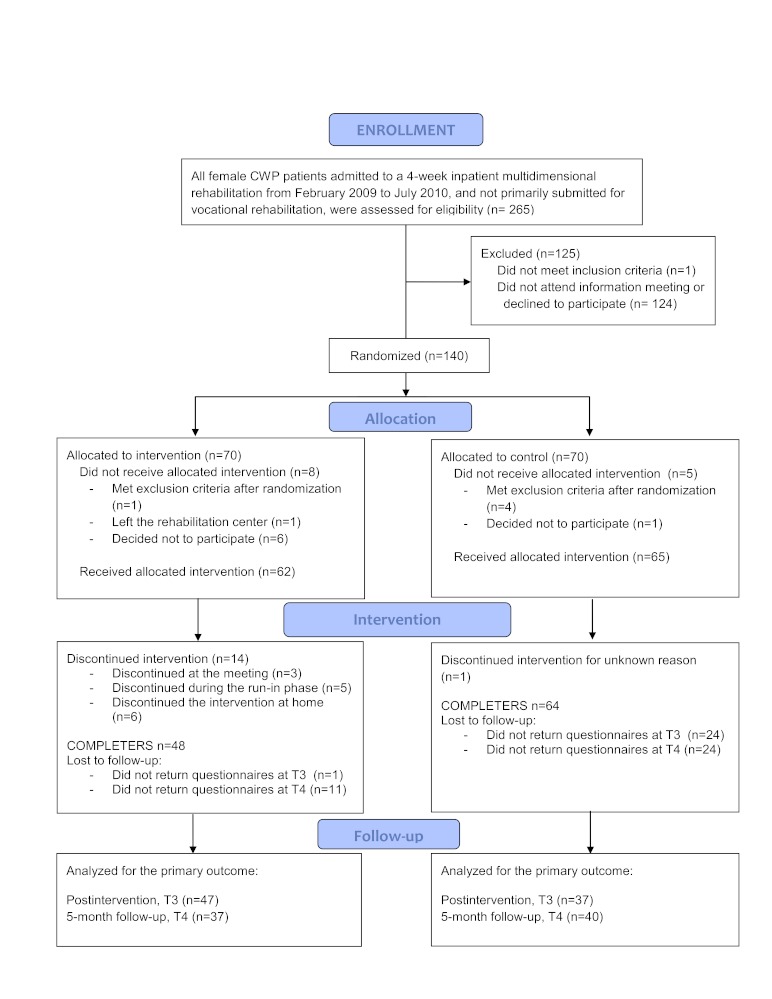
Study design and participant flow.

The overall study design is shown in Figure 1. The design is a parallel-group, randomized controlled trial. Block randomization was used for practical reasons to ensure similar numbers in each group at each time point. All participants attended a 4-week inpatient multidimensional rehabilitation program for chronic pain (see Treatment Procedures). In the fourth week of the program, participants were randomly assigned to 1 of the 2 study groups. The intervention group received a smartphone intervention for 4 weeks after completing the inpatient rehabilitation. Both groups were given access to a noninteractive website with self-help pain management material. Self-reported assessments were gathered at 4 time-points: before (T1) and after (T2) the inpatient program, 4 weeks after discharge when the intervention group had completed their smartphone intervention (T3), and 6 months after discharge from the rehabilitation center (T4). The first 2 questionnaires were received and completed at the rehabilitation center and the last 2 were completed at home and returned by mail. One reminder letter was sent followed by a phone call from a researcher if the questionnaire was not returned.

### Participants

Participants were recruited consecutively from Jeløy Kurbad Rehabilitation Center in Moss, Norway. Patients were referred to the center by their general practitioner or a medical specialist. The inclusion criteria were: female, 18 years or older, participating in the inpatient multidimensional rehabilitation program for chronic pain, having chronic widespread pain for more than 6 months (with or without a diagnosis of fibromyalgia), not participating in another research project at the rehabilitation center, being able to use a smartphone, and not being diagnosed with a profound psychiatric disorder. The study took place between February 2009 and August 2010.

### Ethical Aspects

The study was approved by the Regional Ethics Committee in South-East Norway and by the Norwegian Social Science Services. All participants signed an informed consent form. The study is registered at ClinicalTrial.gov (NCT01236209).

### Procedures

At admission to the inpatient rehabilitation program, all chronic widespread pain patients received a written invitation to attend an informational group meeting where a researcher or a research assistant presented the study. Those who were interested in participating and met the inclusion criteria were given an informed consent form to sign.

A computer-generated sequence list with the 2 groups randomized in blocks of 4 was used because admission of 4 patients per week was expected. The computer-generated inclusion pattern was either 2 participants in each group or 3 to one group, sometimes 3 in the control group and other times 3 in the intervention group, until the final number of 140 was reached. A research assistant put the allocation information in sequentially numbered envelopes and sealed them. A researcher subsequently gave each participant a number and opened the matched envelope to reveal the group allocation. The information about group allocation was revealed to the participant at the inclusion meeting with a nurse in the final week of the inpatient program.

### Assessment Measures

Participants completed self-administered questionnaires in paper format on arrival at the rehabilitation center (T1), at discharge (T2), immediately after the smartphone intervention (T3), and 6 months after discharge from the rehabilitation center (T4), which was 5 months after the smartphone intervention.

The pain catastrophizing scale (PCS) [25] was used to measure the primary outcome variable of the study, catastrophizing. It is a 13-item questionnaire with questions on helplessness, magnification, and rumination. Patients rate items on a scale from 0 (not at all) to 4 (all the time). The total score range for the PCS is 0 to 52, with higher scores reflecting higher degrees of catastrophizing. In our sample, the internal consistency was high on all assessments (Cronbach alpha range .892 to .942). As in prior research, scores greater than 24 were considered high [25,37].

The chronic pain acceptance questionnaire (CPAQ) [38] was used to measure acceptance. It is a 20-item self-report instrument developed to capture the extent of participation in daily activities despite pain and willingness to experience pain without trying to control, alter, or avoid it. It is scored on a 7-point Likert scale (0 = never true; 6 = always true) to give the total score (0-120). Higher scores reflect higher acceptance of pain and higher activities engagement. The reliability of the CPAQ has been established [38]. In our study, the Cronbach alpha coefficients were .814 to .910.

The questions from the 12-item General Health Questionnaire (GHQ) were used [39] with modified response alternatives. Responses to all items were given on the same 4-point scale (much less than usual, same as usual, more than usual, and much more than usual), but not on 2 scales as in the original. The questions measure changes in emotional distress over the previous couple of weeks. A bimodal scoring method was used (1 = symptom present more than usual; 0 = symptom present less than or as usual). Total score range is 0 to 12; indicating the number of symptoms present more than usual during the past 2 weeks. In the current study, the Cronbach alpha coefficients were .703 to .871.

The Chronic Pain Values Inventory (CPVI) is a 12-item measure of importance and success in living according to one’s own values in 6 domains (family, intimate relationships, friendship, work, health, and personal growth) [40]. Each item is rated on a scale from 0 to 5, with higher numbers indicating more importance or success. The mean success rating was used as a measure of values-based action (score range 0-5), as suggested by the authors [40]. In the present study, the Cronbach alpha coefficients for the success scale were .754 to .882.

The current levels (past couple of days) of pain, fatigue, and sleep disturbance were assessed on visual analog scales (VAS) from 0 (no pain/fatigue/sleep disturbance) to 100 (worst imaginable pain/fatigue/sleep disturbance) because these are cardinal symptoms of chronic widespread pain and fibromyalgia.

The original version of the Fibromyalgia Impact Questionnaire (FIQ) was used to measure the impact of fibromyalgia on functioning and symptom levels the past week. It consists of 10 questions with different response alternatives. One question includes 10 subitems related to the ability to perform activities of daily living. The response alternatives are given on a 4-point scale. The other questions enquire about general well-being, ability to work, and level of pain, fatigue, stiffness, and symptoms of anxiety and depression. Questions on symptom level are answered using a VAS from 0 to 100 (high symptom level). The score range is 0 to 100; higher scores indicate greater impairment [41]. The Cronbach alpha coefficients were .807 to .860.

The Short-Form Health Survey (SF-8) was also used to measure functioning. The SF-8 includes 8 items, scored on 5- or 6-point Likert scales, regarding level of functioning the past week. Summary measure scales for the mental health component and the physical component were obtained by using SF-8 Scoring Software 4.5 [42]. Scoring is standardized using the means and standard deviations from a survey from the general adult population in the United States (standardized mean 50, SD 10). Higher scores indicate better functioning; scores above 50 indicate functioning above the average in the US population. In the Norwegian version used in the present study, wording of response options for 2 items differed slightly from the original. In the original, the response alternatives for the item on role physical are none at all, a little bit, some, quite a lot, and could not do daily work. In our version, instead of “a little bit” the response was “very little.” In the original, the response alternatives for the mental health item are not at all, slightly, moderately, quite a lot, and extremely. In our version, “very little” was used instead of “slightly.” The Cronbach alpha coefficients were .785 to .865 in the present study. Use of the noninteractive website was assessed with a self-report 4 weeks after discharge (T3) on how often the participant had visited the website.

Feasibility of the smartphone intervention was assessed with single questions postintervention (T3). For example, “I feel it has been a burden to participate in this intervention (to fill out diaries and receive feedback)” with a 5-point Likert scale (1 = agree completely; 5 = disagree completely).

### Treatment Procedures

#### Inpatient Multidimensional Rehabilitation

All participants participated in a 4-week inpatient multidimensional rehabilitation program for patients with chronic pain. It included education in pain mechanisms and CBT-based pain management (approximately 20 hours), group sessions based on motivational interviewing (4 hours), various forms of aerobic exercise (outdoors, in the pool, and in the gym), stretching, and relaxation. In addition, individual myofascial pain treatment was given in accordance with the protocol of Travell [43,44] and medication was administered as needed (see [9] for details of the program).

#### Smartphone Intervention: Diaries and Daily Situational Feedback

The intervention was developed in 2008. One of the authors (EE) was responsible for the software development. The usability of the intervention was tested in a pretrial study with 6 women with chronic pain. Participation was experienced by the majority as supportive and motivating [20]. The key ACT concepts and a summary of their operationalization in the intervention are shown in Table 1 [28,34].

**Table 1:** Examples of Acceptance and Commitment Therapy (ACT) elements in diaries and feedback.

ACT element	Aim of diaries	Examples of diary questions	Aim of feedback	Examples of feedback
Cognitive defusion/ mindfulness	Awareness supported by making diary entries on thoughts, feelings and behavior three times a day	(1) Right now, my breathing is deep and relaxed. (2) Right now, I believe it is harmful for me to use my body. (3) Right now, I am coping well with the pain.	Reflection on effects of thoughts and feelings on behavior	I see that you register that your breathing is not relaxed. Can you give yourself a minute or two to just notice your breathing? Maybe you can find a quiet spot and close your eyes. You could try breathing deeply and slowly a couple of times. Try focusing only on your breath. If you want, you can listen to the instructions to a short mindfulness breathing exercise on the smartphone/website. All the best, Ann.
Values and values-based action	Awareness, planning and evaluation supported by keeping a diary	Today, I plan to [multiple choices possible]: take a walk/work/rest lying down/do household chores/do relaxation exercises/take care of children or others/eat regularly/exercise at a moderate tempo/do my stretching exercises/spend time with family/rest sitting down/spend time with friends/do some shopping/do aerobic exercises/do something just for the pleasure of it.	Reflection on values and values-based behavior based on reports in diaries	I see you have done your stretching exercises today despite reporting a pain level of 6 (scale from 0 to 10; 0=no pain, 10=worst imaginable pain). Can you give yourself a moment to reflect on why this is something you value and choose to do? I would like to ask you to reflect again on your values, if you are willing to, over the next few days. Values are qualities we ourselves think are important and can give us a sense of direction in life. We can ask ourselves questions like: What kind of a person would I like to be in my relations with my family? What can I do today that would get me a bit closer to this ideal? Is this something I am willing to do? Our values are something we can continuously work toward (like being a caring friend), not something we will obtain once and for all. Have a nice weekend, Ann.
Acceptance vs avoidance	Awareness of a spectrum of pain-related thoughts, feelings, and behavior supported by keeping a diary	(1) Right now, I am afraid to be active because of my pain.” (2) Right now, I feel my life is good despite my pain. (3) Right now, I am doing what I want to even if it means increased pain.	Supporting willingness to act in accordance with values despite pain or discouraging thoughts and feelings	(1) I see that today you are not too pleased with your life. Can you give yourself a moment and reflect on what you would want to do today if you were pain free? Is it possible for you to take a small step toward what you want even with your pain? Could you, instead of saying, “I want this, BUT I have pain and therefore can’t” say “I experience pain AND I am taking baby steps toward something valuable to me.” Are you willing to take small steps? (2) Last night you reported a pain level of 8 and that you felt relaxed, grateful, and pleased with the day’s activity level. Can you take a moment to reflect on what kind of self-management strategies you used yesterday? All the best, Ann

**Figure 2:**
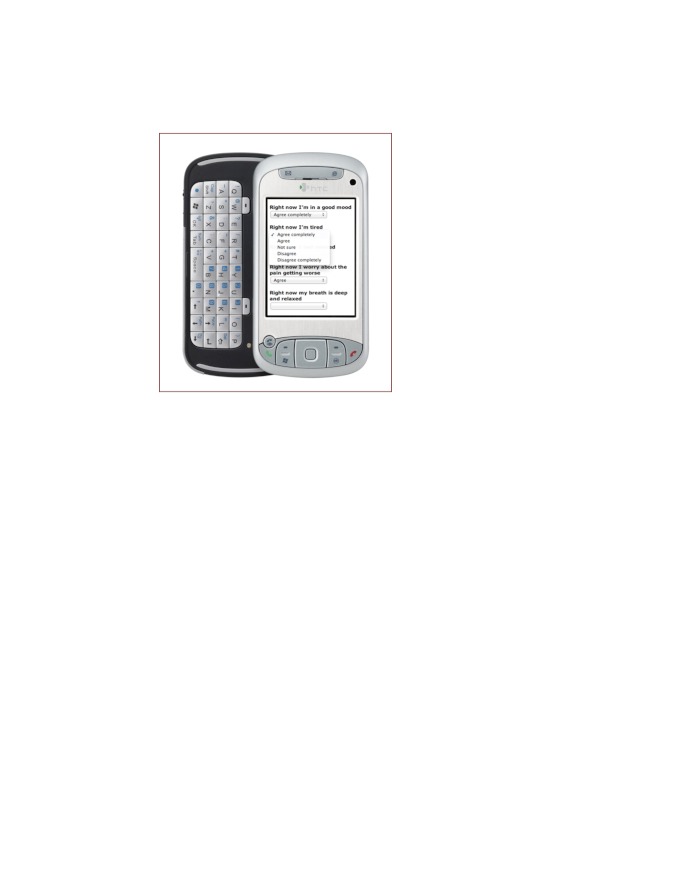
Smartphone screen display of diary.

The smartphone intervention had the following 4 components:

1. Face-to-face session. The intervention started with a 1-hour individual session between a nurse working on the project and the participant. The session took place in the final week before discharge. Each participant was informed about the intervention and asked about functioning, goals for health-related behavior, and support needs. Values and values-based activities were discussed and the patient received 2 written values-based exercises to take home. The participant was lent a smartphone (HTC TyTN) with a touchscreen and a keyboard. The participants received information (name and qualifications) about their therapist for the intervention (in some cases this was the nurse at the meeting). The nurse attending the face-to-face session summarized the meeting and sent it to the relevant therapist.

2. Web-based diaries. The participant was asked to complete 3 diary entries per day using the smartphone. See Figure 2 for a view of the screen display. The diaries included 16 to 24 questions about the current level and interference of pain, and feelings and thoughts related to avoidance, catastrophizing, and acceptance. They also included questions about planned and previous use of self-management activities and daily values-based and practical activities. Lists of self-management activities (eg, mild exercise, stretching, resting, aerobic exercise, and pleasurable activity) were provided as a reminder. The questions were chosen to support self-monitoring and reflection and were formulated in accordance with the experience sampling method principles designed to capture experience in real time without retrospective bias (eg, “Right now I am feeling...”) [45]. See Table 1 for examples of the questions. Participants answered most questions by choosing predefined alternatives or scoring on 5-point Likert scales as shown in Figure 2. All diaries included a comment field giving participants the opportunity to write a short personal message to the therapist. The morning and evening diary entries were sent at fixed hours chosen by each participant. The second diary entry of the day was sent at a time randomly chosen by the Web server, between 11 am and 2 pm. The purpose of including 3 diary entries, including 1 at a randomly chosen time, was to encourage self-monitoring and reflection at different hours and in different situations. At the time scheduled for diary completion, the participant received a Short Message Service (SMS) message with a link to a secure website, where the diary could be opened and questions answered and posted. The participants completed the first diary entry during the face-to-face session, and continued during the final week before discharge with the goal of getting used to the diaries before discharge (a run-in period). After discharge, the diaries were received for 4 weeks. The participant could call a member of the research group (OBK or HE) for technical support. No data were kept on the mobile phone. Up to two automated SMS reminders were sent, if the participant had not responded within 20 to 40 minutes after receiving the SMS signalizing a diary form.

3. Written situational feedback. For 4 weeks after discharge, excluding weekends, participants received daily written feedback from a therapist on the information they had provided in their diaries. The feedback was personalized according to each participant’s situation as reported in the diary. It was written in an empathic style and included repetition of content reported in the diaries, positive reinforcement, reminders of self-management information given at the rehabilitation center, ACT exercises, and reflective questions. The aim was to encourage nonjudgmental awareness of catastrophizing and to stimulate mindfulness and willingness to engage in meaningful activities despite pain or other discouraging intrusions (Table 1). The instructions for the exercises were written directly in the feedback or the participant was referred to exercises available on the mobile phone and/or the website. The feedback was also personalized according to the summary of personal information given at the face-to-face session (eg, family situation and health-related goals) and results on self-reported discrepancy between values and values-based living assessed with the CPVI at the end of the rehabilitation program. The feedback was usually available for the participant within 90 minutes of completing the second diary of the day. If this diary was not submitted, feedback based on information from the most recent submitted diary was sent. When the feedback was available, the participant received an SMS with a link to the website where the feedback could be found. There was no limitation on the length of the feedback, which ranged from a few sentences to a few paragraphs.

The feedback was written by any of 3 of the authors (OBK, TLS, and HE); each participant received signed feedback from the same person throughout the intervention. All therapists had a background in health care sciences (nursing and/or psychology) and had received training in ACT. The feedback protocol was based on ACT for chronic pain [28,34] with a different focus during each of the 4 weeks. For example, in the first week, the focus was on supporting the participant to continue doing the exercises/stretches as recommended at the inpatient program, and during the second week, simple mindfulness exercises were introduced (eg, a few minutes of focused breathing). Once a week, the feedback included an invitation to a values reflection exercise, and every week, questions were included to stimulate reflection on health-related goals. The final feedback comprised a written summary of the registered diary information during the 4-week period. Content from the growing bank of feedback written by all the therapists was used for other participants when appropriate according to the registered information. It took 10 to 15 minutes, on average, to write each piece of feedback. Two members of the group supervised the content of the feedback. They had extensive experience in teaching mindfulness meditation (HE) and supervising CBT/ACT (EAF).

4. Audio files with guided mindfulness exercises. Four audio files with mindfulness exercises (eg, focused breathing) guided by the authors were available on the smartphones.

#### Informational Website With Self-help Pain Management Material

All participants received access to a website with information on self-management strategies for people with chronic pain; not anticipated to have large effect on the study outcomes on its own. It was noninteractive (ie, participants could not register any information or receive feedback). The website included a few written ACT exercises and audio files with mindfulness exercises (as described previously). An example of the written exercises is a behavior analysis aiming to strengthen the ability to observe thought content, feelings, and behavior and the connection between these (adapted from [28]).

### Statistical Procedures

Power analyses were based on the level of reported catastrophizing in chronic widespread pain samples [5,20,46,47], a moderate effect size (Cohen’s *d*=0.5), and allowing for attrition commonly seen in studies on Internet interventions [15,48]. A sample size of 70 participants per group was needed to detect a moderate effect size in the primary outcome variable with a 2-sided 5% significance level and 80% power. To investigate differences in demographic variables and baseline characteristics, independent sample *t* tests, nonparametric tests, and Chi-square tests were used. Data were checked for normal distribution; *t* tests were used when found suitable for parametric analyses, otherwise nonparametric tests (Mann-Whitney) were applied. The Cohen’s *d* effect sizes were calculated by using the difference between the groups’ means divided by the mean standard deviation of both groups. If 1 or 2 items were missing on the GHQ, they were scored as 0 (symptom present less than or as usual). If another instrument included 1 or 2 missing items, the item(s) were replaced with the mean of other items from the participant’s instrument. If 2 response alternatives were marked, the healthier option was chosen. Total score was not computed if more than 2 items were missing, and the case was categorized as missing a total score for the instrument. The number of participants included in each analysis is given. In the intention-to-treat analysis, the last observed value was carried forward when data was missing. Five of the participants who withdrew from the smartphone intervention sent in questionnaires at T3 and at the 5-month follow-up (T4). The intention-to-treat analysis included all participants except those who met the exclusion criteria after randomization (n=135). In the analysis of secondary outcomes, only those who completed the interventions were included (n=112). A significance level of *P*<.05 was used and a tendency toward difference was defined as *P*<.1. Effect sizes were categorized as small (< 0.5), medium (0.5-0.8), and large (> 0.8) in accordance with Cohen [49].

## Results

### Participants

A total of 265 women eligible for the study were invited to an informational meeting about the project. Of these, 124 did not attend the meeting or declined to participate. Only 1 was excluded because of a severe psychiatric disorder. One hundred and forty were randomized to the 2 study arms (Figure 1). Five participants met the exclusion criteria after randomization (they were originally submitted for vocational rehabilitation and included in another research project) and 8 discontinued participation before receiving the allocated intervention. In the intervention group, 14 participants did not complete the intervention. Many of those who discontinued participation did so either at the meeting were the allocation information was given or during the intervention’s run-in period at the rehabilitation center. The most common reason for withdrawal was finding the participation too stressful in combination with the inpatient program. Another 6 participants discontinued the intervention after discharge from the inpatient program. Demographic data and baseline characteristics of the sample by groups are presented in Table 2. Despite randomization, the groups differed in mean pain level (*P*=.02) and physical functioning measured by SF-8 (*P*=.03) at admission to the rehabilitation center. There were no statistically significant differences between the groups at discharge from the rehabilitation center.

**Table 2:** Participants’ characteristics at admission to the inpatient program (T1).

Characteristic		Smartphone intervention (n=69)^a^	Control (n=66)^a^
Age, mean (SD), n		44.59 (11.13), 69	43.80 (11.20), 65
**Marital status, n (%)**			
	Married or cohabiting	42 (60.9)	45 (68.2)
	Divorced	9 (13.0)	6 (9.1)
	Single	13 (18.8)	10 (15.2)
	Widow	4 (5.8)	2 (3.0)
	Unknown	1 (1.4)	3 (4.5)
**Years of education, n (%)**			
	10 years (elementary)	13 (18.8)	8 (12.1)
	11-13 years (high school)	19 (27.5)	30 (45.5)
	>13 years (college/university)	30 (43.5)	23 (34.8)
	Unknown	7 (10.1)	5 (7.6)
**Employment status, n (%)**			
	Working/studying	15 (21.7)	8 (12.1)
	Unemployed	3 (4.3)	1 (1.5)
	On sick leave	27 (39.1)	34 (51.5)
	On disability pension	12 (17.4)	13 (19.7)
	Working/studying part time and part time sick leave	8 (11.6)	5 (7.6)
	Other combination of the above	4 (5.8)	4 (6.1)
	Unknown	0	1 (1.5)
Diagnosed with fibromyalgia, n (%)		55 (80.9)	54 (84.4)
Duration of symptoms (years), mean (SD)		13.11 (8.78)	15.47 (12.09)
**Current VAS** ^b^ **rating (past couple of days), mean (SD), n**			
	Pain	67.08 (17.47), 69	57.85 (21.60), 66
	Fatigue	67.40 (23.73), 69	64.72 (21.02), 66
	Sleep disturbance	57.24 (26.22) 68	55.16 (23.38), 66
**Assessments** ^**c**^ **and ranges, mean (SD), n**			
	PCS (0-52^d^)	21.24 (10.33), 63	20.80 (9.45), 62
	CPAQ (0^d^-120)	56.48 (15.02), 58	53.87 (13.81), 57
	FIQ (0-100^d^)	58.75 (16.39), 69	58.58 (16.04), 66
	SF-8, physical (0^d^-100)	31.91 (7.57), 65	34.75 (7.35), 62
	SF-8, mental (0^d^-100)	39.33 (10.49), 65	39.34 (9.61), 62
	GHQ-12 (0-12^d^)	3.32 (3.38), 62	3.02 (3.38), 61
	CPVI (0^d^-6)	2.07 (0.95), 64	2.01 (0.73), 61

^a^ Patients meeting exclusion criteria after randomization were not included in this analysis.

^b^ VAS: visual analog scale, range 0-100.

^c^ PCS: pain catastrophizing scale; CPAQ: Chronic Pain Acceptance Questionnaire; FIQ: Fibromyalgia Impact Questionnaire; SF-8: Short-Form Health Survey; GHQ-12: General Health Questionnaire; and CPVI: Chronic Pain Values Inventory.

^d^ Values that indicate maximum symptom scores/least health.

Information about fibromyalgia diagnosis was available for 132 participants, and 82.6% of these met the American College of Rheumatology’s classification criteria for fibromyalgia. As shown in Tables 3 and 4, no significant group differences were detected at discharge from the rehabilitation center on any of the outcome variables.

**Table 3:** Means and standard deviations for the primary outcome measure, the pain catastrophizing scale (PCS), at admission to inpatient rehabilitation (T1), at discharge (T2), immediately after intervention (T3), and 5 months after the intervention period (T4).

PCS		T1	T2	T3	T4
**ITT and LOCF** ^**a**^ **, mean (SD), n**					
	Intervention	21.24 (10.33), 63	16.06 (10.37), 68	12.32 (9.22), 69	13.59 (9.72), 69
	Control	20.80 (9.45), 62	15.33 (9.31), 65	16.07 (9.48), 65	17.43 (11.60), 66
**Per protocol, mean (SD), n**					
	Intervention	20.56 (10.08), 43	14.61 (8.93), 45	9.20 (5.85), 47	10.92 (8.58), 37
	Control	20.78 (9.59), 60	15.46 (9.76), 57	15.71 (9.11), 37	18.70 (12.45), 40
**PCS score > 24, n (%)**					
	Intervention	13 (30.2)	7 (15.6)	0 (0)	1 (2.7)
	Control	20 (33.3)	10 (17.5)	6 (16.7)	14 (35.0)

^a^ ITT: intention-to-treat; LOCF: last observation carried forward.

**Table 4:** Means and standard deviations for the secondary outcome measures at admission to the inpatient rehabilitation (T1), at discharge (T2), immediately after intervention (T3) and 5 months after the intervention period (T4) for the participants who completed the study.

Secondary outcome measures^a^		T1 mean (SD), n	T2 mean (SD), n	T3 mean (SD), n	T4 mean (SD), n
**CPAQ**					
	Intervention	56.45 (15.22), 40	62.00 (13.62), 44	72.50 (15.67), 44	71.42 (18.38), 36
	Control	53.94 (13.92), 56	62.21 (10.15), 57	63.55 (13.33), 38	62.47 (14.87), 38
**FIQ**					
	Intervention	58.46 (17.26), 48	46.38 (16.92), 47	49.12 (19.65), 47	46.45 (19.37), 37
	Control	58.35 (16.18), 64	49.10 (17.32), 62	53.07 (18.68), 39	59.92 (16.46), 40
**SF-8, physical**					
	Intervention	32.12 (7.74), 45	36.68 (8.42), 40	35.24 (8.74), 46	37.54 (9.44), 37
	Control	34.98 (7.13), 60	35.86 (8.24), 49	36.55 (8.17), 37	34.37 (8.59), 40
**SF-8, mental**					
	Intervention	39.50 (10.67), 45	45.70 (8.06), 40	46.82 (8.85), 47	44.34 (10.42), 37
	Control	39.09 (9.61), 60	44.83 (9.69), 49	41.01 (9.70), 37	39.78 (10.70), 40
**GHQ-12**					
	Intervention	3.19 (3.21), 43	1.20 (2.02), 45	1.78 (2.51), 46	1.89 (2.57), 37
	Control	2.97 (3.43), 59	0.63 (1.01), 57	1.86 (2.07), 37	2.85 (3.25), 40
**CPVI**					
	Intervention	2.05 (0.95), 44	2.47 (0.91), 46	2.95 (0.99), 46	2.62 (0.93), 37
	Control	2.02 (0.74), 59	2.52 (0.68), 54	2.35 (0.91), 38	2.27 (0.83), 40
**Pain, VAS**					
	Intervention	66.59 (17.58), 48	53.07 (22.20), 47	54.14 (24.06), 47	51.96 (23.76), 37
	Control	57.32 (21.56), 64	52.99 (21.27), 61	50.56 (23.37), 40	58.45 (22.46), 40
**Fatigue, VAS**					
	Intervention	69.29 (23.98), 48	51.38 (27.75), 47	52.26 (29.18), 47	55.24 (25.73), 37
	Control	64.08 (21.01), 64	50.10 (24.28), 61	53.20 (24.04), 40	65.03 (21.64), 40
**Sleep disturbance, VAS**					
	Intervention	54.77 (26.99), 47	43.97 (25.77), 47	43.41 (30.60), 47	43.32 (27.88), 37
	Control	54.59 (23.31), 64	48.12 (24.57), 62	48.90 (26.12), 40	57.68 (24.67), 40

^a^ CPAQ: Chronic Pain Acceptance Questionnaire; FIQ: Fibromyalgia Impact Questionnaire; SF-8: Short-Form Health Survey; GHQ-12: General Health Questionnaire; CPVI: Chronic Pain Values Inventory; and VAS: visual analog scale.

### Within-Group Analysis

Temporal changes within groups and effect sizes within the groups are presented in Tables 5 and 6. Analysis according to the intention-to-treat principles showed a small positive effect on catastrophizing in the intervention group at both assessments and a small negative effect was shown at the 5-month follow-up in the control group. Per-protocol analysis revealed moderate effects on catastrophizing, pain acceptance, and success in living according to values in the intervention group immediately after the follow-up intervention period. The control group did not improve on these variables. The percentage of participants with a total score above 24 on the PCS decreased in the smartphone group, but not in the control group. Increased emotional distress was reported in the control group at 5-month follow-up. Function and symptom impairment, as measured by the FIQ, was increased at both measurements in the control group only. Six months after discharge from the rehabilitation center (5-month follow-up, T4), the improvement in catastrophizing and pain acceptance remained for the intervention group. The changes in success in values-based living were not maintained. However, the control group reported less success in values-based living at the 5-month (T4) follow-up compared to the level at discharge. Pain level was stable in both groups. Fatigue had increased in the control group at the 5-month follow-up and there was a tendency toward more sleep disturbance, which was not seen in the intervention group. Factor analysis of the Norwegian version of the CPAQ revealed some inconsistencies with the 2-factor structure of the scale; 4 items were found to not fit the originally described structure [38]. Because we do not report on the questionnaire’s subscale, we decided to include all questions in our analysis. The results did not differ in a significant way when the 4 items were excluded.

**Table 5:** Mean differences for the primary outcome measure, the pain catastrophizing scale (PCS) within groups, confidence intervals (CI), and effect sizes (ES).

PCS		Mean difference T2–T3^a^ (n)	95% CI T2–T3^a^	Mean difference T2–T4^b^ (n)	95% CI T2–T4^b^	ES T2–T3^a^	*P* value^c^	ES T2–T4^b^	*P* value^c^
**ITT and LOCF** ^**d**^									
	Intervention	–3.65 (68)	–5.24 to –2.07	–2.37 (68)	–4.32 to –0.41	0.37	< .001	0.24	.02
	Control	0.74 (65)	–0.70 to 2.17	2.30 (65)	0.43-4.16	–0.08	.31	–0.22	.02
**Per protocol**									
	Intervention	–5.09 (44)	–7.00 to –3.18	–2.96 (36)	–5.78 to –0.13	0.69	< .001	0.33	.04
	Control	1.67 (34)	–1.06 to 4.40	2.58 (37)	–0.37 to 5.53	–0.18	.22	–0.24	.09

^a^ T2: at discharge; T3: immediately after intervention.

^b^ T2: at discharge; T4: 5 months after intervention.

^c^ *P* values for paired samples *t* tests.

^d^ ITT: intention-to-treat; LOCF: last observation carried forward.

**Table 6:** Mean differences for the secondary outcome measures within groups, confidence intervals (CI), and effect sizes (ES) for the completers.

Secondary outcome measures^a^		Mean difference T2–T3^b^ (n)	95% CI T2–T3^b^	Mean difference T2–T4^c^ (n)	95% CI T2–T4^c^	ES T2–T3^a^	*P* value^d^	ES T2–T4^c^	*P* value^d^
**CPAQ**									
	Intervention	8.75 (40)	5.96-11.54	7.29 (34)	3.11-11.47	0.58	< .001	0.45	.001
	Control	0.69 (36)	–2.90 to 4.29	0.40 (35)	–3.43 to 4.23	0.06	.70	0.03	.83
**FIQ**									
	Intervention	3.10 (46)	–1.01 to 7.20	1.60 (36)	–4.40 to 7.60	–0.17	.14	–0.09	.59
	Control	6.61 (38)	2.14-11.09	10.46 (39)	6.43-14.49	–0.36	.005	–0.62	< .001
**SF-8, physical**									
	Intervention	–1.69 (39)	–3.96 to 0.59	0.06 (30)	–3.73 to 3.86	–0.19	.14	0.01	.97
	Control	–1.17 (29)	–4.18 to 1.83	–2.41 (32)	–5.17 to 0.36	–0.14	.43	–0.29	.09
**SF-8, mental**									
	Intervention	1.28 (39)	–1.72 to 4.28	–1.51 (30)	–4.93 to 1.92	0.15	.39	–0.17	.38
	Control	–3.59 (29)	–7.04 to –0.14	–4.92 (32)	–9.55 to –0.30	–0.37	0.04	–0.50	.04
**GHQ-12**									
	Intervention	0.58 (43)	–0.06 to 1.22	0.80 (35)	–0.42 to 2.02	–0.25	.07	–0.34	.19
	Control	1.26 (34)	0.59-1.94	2.38 (37)	1.12-3.63	–0.80	.001	–1.09	< .001
**CPVI**									
	Intervention	0.49 (44)	0.26-0.72	0.15 (36)	–0.11 to 0.42	0.52	< .001	0.16	.25
	Control	–0.22 (33)	–0.49 to 0.06	–0.47 (34)	–0.83 to –0.10	–0.28	0.12	–0.63	.01
**Pain, VAS**									
	Intervention	1.11 (46)	–3.94 to 6.16	0.61 (36)	–7.03 to 8.24	–0.05	.66	–0.03	.87
	Control	–0.99 (38)	–7.48 to 5.50	5.82 (38)	–1.26 to 12.90	0.04	.76	–0.26	.10
**Fatigue, VAS**									
	Intervention	1.13 (46)	–4.94 to 7.21	7.73 (36)	–2.26 to 17.72	–0.04	.71	–0.29	.13
	Control	5.44 (38)	–1.13 to 12.01	12.15 (38)	6.29-18.00	–0.22	.10	–0.51	< .001
**Sleep disturbance, VAS**									
	Intervention	–0.14 (46)	–7.03 to 6.76	2.15 (36)	–7.81-12.12	0.01	.97	–0.08	.66
	Control	3.96 (39)	–5.42 to 13.33	7.66 (39)	–0.62 to 15.95	–0.15	.40	–0.30	.07

^a^ CPAQ: Chronic Pain Acceptance Questionnaire; FIQ: Fibromyalgia Impact Questionnaire; SF-8: Short-Form Health Survey; GHQ-12: General Health Questionnaire; CPVI: Chronic Pain Values Inventory; and VAS: visual analog scale.

^b^ T2: at discharge; T3: immediately after intervention.

^c^ T2: at discharge; T4: 5 months after intervention.

^d^ *P* values for paired samples *t* tests.

### Between-Group Analysis

The between-group effect sizes are shown in Table 7. The intention-to-treat analysis showed a small effect between the groups after the intervention and a tendency (*P*=.05) toward a small effect at 5-month follow-up. The effect size on catastrophizing for completers was large immediately after the intervention period and remained moderate at the 5-month follow-up. Moderate effect sizes were also found for acceptance at both assessments times. There was a moderate effect on values-based living right after the smartphone intervention and a tendency toward moderate effects at 5-month follow-up. A moderate effect on sleep disturbance was found at the 5-month follow-up and a tendency toward moderate effect on fatigue. No effect was found on pain level. A moderate effect was found for functioning and symptom severity measured by the FIQ.

**Table 7:** Between-group effect sizes (ES) after the smartphone intervention (T3) and at 5-month follow-up (T4).

Outcome measure^a^		ES at T3	*P* value^b^	ES at T4	*P* value^b^
**Primary**					
	PCS (ITT and LOCF)	0.40	.01	0.36	.05
	PCS (per protocol)	0.87	< .001	0.74	.003
**Secondary (per protocol)**					
	CPAQ	0.62	.007	0.54	.02
	FIQ	0.21	.35	0.75	.001
	SF-8, physical	–0.15	.64	0.35	.13
	SF-8, mental	0.63	.005	0.43	.06
	GHQ-12	0.03	.56	0.33	.16
	CPVI	0.63	.005	0.40	.08
	Pain, VAS	–0.15	.49	0.28	.22
	Fatigue, VAS	0.04	.87	0.41	.07
	Sleep disturbance, VAS	0.19	.36	0.55	.02

^a^ PCS: pain catastrophizing scale; ITT: intention-to-treat; LOCF: last observation carried forward; CPAQ: Chronic Pain Acceptance Questionnaire; FIQ: Fibromyalgia Impact Questionnaire; SF-8: Short-Form Health Survey; GHQ-12: General Health Questionnaire; CPVI: Chronic Pain Values Inventory; and VAS: visual analog scale.

^b^ *P* values for independent *t* tests or nonparametric tests.

### Withdrawal From Participation

Of the 135 participants, 112 completed the study period (Figure 1). Twenty-one withdrew from the intervention group (30.4%) and 2 withdrew from the control group (3.0%). Because of the small size of the withdrawal group (n=23), group differences with *P*<.2 are described here. The participants who completed the study tended to be younger (mean 43.33, SD 11.18) than the ones who withdrew (mean 48.43, SD 10.06, *P*=.07). There was a tendency toward higher pain level at admission in the group who withdrew (mean 68.79, SD 17.48) than in the group who completed (mean 61.29, SD 20.39, *P*=.15). There was also a tendency toward a higher level of sleep disturbance in the group who withdrew (mean 63.68, SD 23.81) than in the group who completed (mean 54.67, SD 24.81, *P*=.11). A tendency toward a difference was seen in physical functioning as measured by the SF-8; those who withdrew had lower functioning (mean 31.11, SD 7.67) compared to those who completed (mean 33.76, SD 7.50, *P*=.14). At discharge from the rehabilitation center, those who withdrew had more self-reported success in values-based living (mean 2.82, SD 0.91) compared to those who completed (mean 2.50, SD 0.79, *P*=.12).

### Response Rates to Assessment Questionnaires

In accordance with the intention-to-treat principle, the response rate for all included participants was 68.1% at T3 (immediately after the smartphone intervention) and 62.2% at T4 (5-month follow-up). There was a higher response rate in the intervention group (75.4%) than in the control group (60.6%) at T3, but the rates were similar at T4 (63.8% and 60.6%, respectively). When only the completers were included in the analysis, more differences in response rates were found. The response rate for the intervention group was 97.9% at T3 and 77.1% at T4. The response rate was 62.5% in the control group at both T3 and T4. The numbers of participants excluded because more than 2 items were missing varied and the number included in each instrument analysis is shown in Table 3.

Comparison of demographic and outcome variables at baseline (T1) between participants who completed the study and returned questionnaires at T3 (n=87) and those who did not return them (n=25) revealed a few differences. Because of the small size of the group who did not return the questionnaires, group differences with *P*<.2 are described here. Those who did return T3 questionnaires had less function impairment and symptom levels at discharge (mean 46.10, SD 17.18) measured by FIQ compared to those who did not return the questionnaire (mean 54.38, SD 15.58, *P*=.04). The same trend was seen in the results of the physical component of the SF-8; those who did return the questionnaires at T3 had better physical functioning (mean 37.20, SD 8.32) at discharge compared to those who did not return them (mean 32.87, SD 7.38, *P*=.04). Those who completed the study, but did not return the questionnaires at T4 (n=35) had lower scores on the CPVI (success scale) (mean 1.78, SD 0.77) at T1 than those returning the questionnaires (mean 2.16, SD 0.84, *P*=.03). At baseline (T1), those who returned T3 questionnaires had higher pain level (mean 62.84, SD 20.02) than those not returning them (mean 55.90, SD 21.17, *P*=.12). There was a tendency toward higher pain level (mean 63.11, SD 20.06) at T1 in those who returned questionnaires at the 5-month follow-up (T4) than those who did not return them (mean 57.29, SD 20.84, *P*=.16). There was also a tendency toward having experienced pain for longer time (mean 17.28 years, SD 13.51) in those who did not return questionnaires at T4 compared with those who did return them (mean 12.77, SD 9.62, *P*=.07). Also, there was a tendency toward worse physical functioning at discharge (mean 34.24, SD 8.54) measured with SF-8 in those who did not return questionnaires at T4 compared to those who did return them (mean 37.10, SD 8.08, *P*=.14).

### Response Rate to the Smartphone Diary Entries and Experience of Participation

The response rate to the diary entries during the 4 weeks after discharge ranged from 27.4% to 95.2%, with a mean of 68.5% and a median of 70.2%. Most (83.3%) participants received 84 entries (4 weeks). A total of 16.7% received additional days of entries to compensate for holidays to ensure 20 days with registration and feedback. Of the 48 participants who completed the study in the smartphone intervention, 43 reported on the experience of participating. Ten (23.3%) participants agreed somewhat that the participation had been experienced as a burden, 9 (20.9%) were neutral in their opinion, 9 (20.9%) disagreed somewhat to the statement, and 15 (34.9%) totally disagreed with the statement that participation was experienced as a burden. Of those who completed the study, 37 (86.0%) agreed somewhat or totally that participation was useful. Three participants (7.0%) were neutral toward this item, and 3 (7.0%) participants disagreed somewhat or totally that participation was useful.

### Use of the Informational Website

Of the participants who completed the study in the smartphone intervention, 22 (45.8%) reported never visiting the website. Six (12.5%) visited it once, 8 participants (16.7%) viewed it twice, and 11 (22.9%) viewed it three times or more. One participant did not respond to the question. In the control group, 38 participants who completed the study answered the question. Twelve (18.8%) reported never having visited the website, 5 (7.8%) viewed it once, 9 (14.1%) viewed it twice, and 12 (18.8%) visited 3 or more times.

## Discussion

### Principal Results

To our knowledge, this is the first study to investigate the efficacy of a smartphone-delivered intervention aiming to reduce catastrophizing and increase functioning in patients with chronic widespread pain. The results from the per-protocol analysis indicate that this intervention with diaries and written personalized feedback reduced catastrophizing and increased acceptance in women with chronic widespread pain and that these effects persisted 5 months after the intervention. At the 5-month follow-up, the control group experienced increased emotional distress compared to the distress at discharge from the inpatient program, whereas the smartphone group did not. The between-group effect size on functioning and symptom level was moderate (0.75) at the 5-month follow-up measured with the FIQ, but no difference was seen in the physical component of the SF-8. One reason for this may be the general nature of the items in the SF-8 compared to the questions in FIQ, possibly making it less sensitive to changes. The results also show a tendency toward increased improvement in values-based living in the intervention group compared to the control group.

When all randomized participants were included in the analysis, the effect size of catastrophizing was small. This may partly be explained by the higher rate of nonresponse in the control group and the method of carrying the last observed value forward resulting in the possibility of a false positive effect for the control group. Scores above 24 on the PCS have been categorized as indicating a high risk for reduced functioning [37]. None of the 7 participants who exceeded this limit before starting the smartphone intervention did so at the end of the intervention. Only one participant was above this criterion again 5 months later. The opposite trend was seen in the control group; an increased number of participants were classified as “catastrophizers.”

The intervention was based on CBT, one of the most commonly used models of change in Internet intervention research [50]. We used CBT-related ACT, in which the goal is not to change or reconstruct the content of thoughts, but rather to change how it influences behavior. Behavior change is supported when patients learn to mindfully observe and accept inner experiences and to commit to values-based activity despite challenging thoughts, feelings, or symptoms [28,29,51]. By doing this, the influence of catastrophizing thoughts is expected to be diminished, but by a process other than that described in more traditional CBT, where problematic thoughts are more rationally challenged [28,52]. The reduction in catastrophizing and the increase in acceptance support previous studies that show negative correlations between mindfulness and acceptance, and catastrophizing [37,53,54]. The changes in catastrophizing, acceptance, and functioning in those who completed the study cannot be attributed to changes in levels of pain, or vice versa, because no significant reduction in pain level was found. This is in line with the findings of a recent randomized controlled trial in which fibromyalgia patients who had participated in a 12-week group-based ACT reported more improvement of the condition compared to a waiting-list control group despite no changes in pain level [55]. However, our results differ from that of other previous studies—a small effect size on pain intensity was found in a meta-analysis including 9 randomized trials of acceptance-based interventions [56]. This finding may be explained in part by the fact that the present intervention follows another intervention that had reduced the pain level. The control group showed an increased level of fatigue and a tendency toward an increase in sleep disturbance at the 5-month follow-up. This may indicate that the follow-up intervention might have contributed to the prevention of sleep disturbance. A positive correlation has been found between psychological flexibility and improved sleep quality in people with chronic pain [57].

### Strengths and Limitations

To our knowledge, no randomized studies on smartphone interventions based on ACT have been previously published. Our results may support the notion that ACT can be successfully used as a framework for smartphone interventions with mainly written communication. This also supports the results of two recent studies of interventions that provided written ACT-based self-help material and weekly telephone support from a therapist, for 6 and 7 weeks, for patients with chronic pain. Both studies found medium to large effect sizes on pain acceptance [58,59]. However, the present intervention contained many possibly active components and the study design did not allow for any distinction between possible mechanisms and explanations. It is possible that the intervention group benefited from having higher expectations of improvement and from the empathic attention and encouragement from a health care provider [50]. As stated in the guidelines for Internet intervention research, it may still be premature to require demonstration of processes of change in Internet interventions because of the newness of the field [50]. Our results are consistent with the findings of a study that tested the efficacy of a similar mobile phone-delivered intervention with diaries and daily CBT-based feedback for patients with irritable bowel syndrome. The intervention reduced catastrophizing thoughts and the effects were maintained at a 3-month follow-up [18]. Our results are also in line with the results of a follow-up telephone intervention for chronic pain patients designed to support self-monitoring, to give a review of learned self-management techniques, and to provide monthly feedback from a CBT therapist after 11 weeks of group CBT. The intervention was found to reduce pain catastrophizing [13].

Studies on Internet-based interventions and interventions using SMS to support self-management of chronic illness show promising results [60-62]. A review of 14 studies that used SMS to support health behavior change included 6 randomized controlled trials. The duration of the interventions varied from 6 weeks to 1 year and the frequency of communication ranged from many times daily to less than monthly. All but one were effective in supporting positive behavior change, with effect sizes ranging from small to large. However, follow-up data was limited [60]. Reviews of Internet-based interventions for patients with various chronic pain conditions indicate a positive effect on pain, but results on psychological outcomes have been inconsistent [14,15,62]. An important feature of a successful therapeutic relationship is the therapist’s ability to respond to what the patient expresses, and tailored or personalized messages have been found to be more effective in supporting behavior change than standardized ones [60]. Our intention was to support a therapeutic relationship with the therapist responding to the expressions made in the diaries and with the goal of sending the individualized feedback as soon as practically possible.

The present study has some limitations. The generalizability of the results is reduced by several factors. Firstly, the intervention group had a withdrawal rate of 30% and this might have resulted in differences in the characteristics of completers between groups. Indeed, there was a trend toward the completers being younger and having less pain, less sleep disturbances, and better function measured with SF-8 at baseline. At admission to the inpatient program (T1), the participants in the smartphone intervention group reported higher pain levels and lower physical functioning compared to the control group. At discharge (T2), this difference was no longer evident. This indicates that participants in the smartphone intervention group improved more on those two variables during the inpatient program compared to the control group. It is possible that this implies some not-assessed differences in the groups’ characteristics. Our intention with a run-in period during the final week of the inpatient program was to give the patients a chance to get used to the smartphone diaries before returning home. However, our results may indicate that this might not have been suitable for all participants because several participants withdrew during the run-in phase; it might have been more feasible to give the participants the choice of starting the intervention after discharge from the inpatient program. During the inpatient program, the participants had a busy schedule with activities and may, therefore, have experienced adding the smartphone diaries as stressful. They chose to receive their morning and evening diaries at hours suitable for their schedule at home, which may possibly have been inconvenient while still at the rehabilitation center. High withdrawal rates have been a challenge in SMS-based and Web-based interventions [60]. In a review of 17 trials of Internet self-management interventions for people with chronic pain, the withdrawal rate ranged from 6% to 59% with a median withdrawal rate of 27% [14]. Therapist contact and tailored or personalized messages have been found to correlate with lower withdrawal rates, but as our results show, other factors clearly also play roles. Despite the high withdrawal rate, most experienced the present intervention as useful. In a qualitative study with 7 of our participants, the intervention was described as motivating and supportive [63].

Secondly, the response rate to assessment questionnaires was below 70% at both follow-ups; this affects the generalizability of the results because data cannot be assumed to be missing at random. The response rate was different between the groups, with a lower response rate in the control group immediately after the intervention period. This is commonly experienced in randomized controlled studies [64]. Those who did not return questionnaires after the intervention period (T3) had lower pain levels at baseline (T1) than those who did. Also, those who did not return questionnaires after the smartphone intervention period (T3) had more function and symptom impairment at discharge from the center compared with those who returned those questionnaires. Since all except 1 participant in the smartphone group returned the questionnaires after the intervention (T3) and those who did not respond belonged to the control group, it may be that the level of functional impairment and symptom severity for the control group was, in fact, higher. The 5-month follow-up results could also be affected because there was a tendency toward those not returning the questionnaires reporting less pain at baseline (T1) and better functioning and less symptom severity at discharge (T2). Finally, the generalizability is also affected by the fact that just over half of those eligible to participate were included in the study. We do not know if those who chose to participate differed in any way from those who declined participation. The introduction meeting for the study was scheduled during the second week of the rehabilitation program. For some it may have been too early to consider involvement in a follow-up intervention and others may have used the opportunity to prioritize private time in the tight rehabilitation schedule instead of listening to study information. Moreover, in the stress management part of the rehabilitation program, the patients were encouraged to set limits and say no to requests they felt added more stress to their everyday burden. Patients with high self-efficacy regarding coping after discharge may have been more likely to not attend the informational meeting. Also, because all those who were eligible for the study received a short information letter about the study, some may have found the intervention format unsuitable. In a future study, this kind of intervention might be made more feasible by adding a virtual social support group including fellow participants from the inpatient program. The increase in function impairment and symptom levels in the control group after discharge is not in line with the results of a study on 200 patients with chronic widespread pain or fibromyalgia participating in the same kind of 4-week inpatient program at the same rehabilitation center. The results of the study showed significant improvements in functioning and symptom levels, maintained at both 6- and 12-month follow-ups [9]. However, the samples were not identical because we have excluded men in the present study and those submitted primarily for vocational rehabilitation. Selection bias in our sample may also have had an impact (ie, those with positive long-term effects may have elected not to participate in the study). Nevertheless, the results of the long-term effects of multidimensional pain programs are inconclusive and the need for maintenance support has been clearly stated [65,8,11,12].

This smartphone intervention was developed in 2008 and was delivered using first-generation smartphones. Today, the diary part of the intervention can easily be converted to a smartphone application. Future research might investigate whether automatic feedback could be effectively tailored to diaries and integrated in an application to reduce the investment of human resources used in the presented intervention.

### Conclusion

Our results give preliminary support to the efficacy of a smartphone intervention for catastrophizing, acceptance, functioning, and symptom level in women with chronic widespread pain. In addition to subgroup analyses of participants and results on long-term effects, research on practice implications, innovation, and added values for the users are needed.

## Multimedia Appendix 1

CONSORT eHealth V1.6.1 [66].

## Abbreviations

ACT: Acceptance and Commitment Therapy
CBT: cognitive behavioral therapy
CPAQ: Chronic Pain Acceptance Questionnaire
CPVI: Chronic Pain Values Inventory
ES: effect sizes
FIQ: Fibromyalgia Impact Questionnaire
GHQ: General Health Questionnaire
LOCF: last observation carried forward
PCS: Pain Catastrophizing Scale
SD: standard deviations
SDT: self-determination theory
SF-8: Short-Form Health Survey
SMS: Short Message Service
VAS: visual analog scale

## References

[1] Croft P, Rigby AS, Boswell R, Schollum J, Silman A (1993). The prevalence of chronic widespread pain in the general population. J Rheumatol.

[2] Clauw DJ, Crofford LJ (2003). Chronic widespread pain and fibromyalgia: what we know, and what we need to know. Best Pract Res Clin Rheumatol.

[3] Shipley M (2010). Chronic widespread pain and fibromyalgia syndrome. Medicine.

[4] Lindell L, Bergman S, Petersson IF, Jacobsson LT, Herrström P (2000). Prevalence of fibromyalgia and chronic widespread pain. Scand J Prim Health Care.

[5] Cöster L, Kendall S, Gerdle B, Henriksson C, Henriksson KG, Bengtsson A (2008). Chronic widespread musculoskeletal pain - a comparison of those who meet criteria for fibromyalgia and those who do not. Eur J Pain.

[6] Flor H, Turk DC (2011). Chronic Pain: An Integrated Biobehavioral Approach.

[7] Goldenberg DL, Burckhardt C, Crofford L (2004). Management of fibromyalgia syndrome. JAMA.

[8] Häuser W, Bernardy K, Arnold B, Offenbächer M, Schiltenwolf M (2009). Efficacy of multicomponent treatment in fibromyalgia syndrome: a meta-analysis of randomized controlled clinical trials. Arthritis Rheum.

[9] Wigers SH, Finset A (2007). [Rehabilitation of chronic myofascial pain disorders]. Tidsskr Nor Laegeforen.

[10] Turk DC, Sherman JJ (2002). Treatment of patients with fibromyalgia syndrome. Psychological Approaches to Pain Management: A Practitioner's Handbook.

[11] Turk DC, Rudy TE (1991). Neglected topics in the treatment of chronic pain patients--relapse, noncompliance, and adherence enhancement. Pain.

[12] Morley S (2008). Relapse prevention: still neglected after all these years. Pain.

[13] Naylor MR, Keefe FJ, Brigidi B, Naud S, Helzer JE (2008). Therapeutic Interactive Voice Response for chronic pain reduction and relapse prevention. Pain.

[14] Bender JL, Radhakrishnan A, Diorio C, Englesakis M, Jadad AR (2011). Can pain be managed through the Internet? A systematic review of randomized controlled trials. Pain.

[15] Macea DD, Gajos K, Daglia Calil YA, Fregni F (2010). The efficacy of Web-based cognitive behavioral interventions for chronic pain: a systematic review and meta-analysis. J Pain.

[16] Buhrman M, Nilsson-Ihrfeldt E, Jannert M, Ström L, Andersson G (2011). Guided internet-based cognitive behavioural treatment for chronic back pain reduces pain catastrophizing: a randomized controlled trial. J Rehabil Med.

[17] Luxton DD, McCann RA, Bush NE, Mishkind MC, Reger GM (2011). mHealth for mental health: Integrating smartphone technology in behavioral healthcare. Prof Psychol-Res Pr.

[18] Oerlemans S, van Cranenburgh O, Herremans PJ, Spreeuwenberg P, van Dulmen S (2011). Intervening on cognitions and behavior in irritable bowel syndrome: A feasibility trial using PDAs. J Psychosom Res.

[19] Sorbi MJ, Mak SB, Houtveen JH, Kleiboer AM, van Doornen LJ (2007). Mobile Web-based monitoring and coaching: feasibility in chronic migraine. J Med Internet Res.

[20] Kristjánsdóttir ÓB, Fors EA, Eide E, Finset A, van Dulmen S, Wigers SH, Eide H (2011). Written online situational feedback via mobile phone to support self-management of chronic widespread pain: a usability study of a Web-based intervention. BMC Musculoskelet Disord.

[21] Rosser BA, McCullagh P, Davies R, Mountain GA, McCracken L, Eccleston C (2011). Technology-mediated therapy for chronic pain management: the challenges of adapting behavior change interventions for delivery with pervasive communication technology. Telemed J E Health.

[22] Rosser BA, Eccleston C (2011). Smartphone applications for pain management. J Telemed Telecare.

[23] Turk DC, Gatchel RJ (2002). Psychological Approaches to Pain Management: A Practitioner's Handbook.

[24] Vlaeyen JW, Linton SJ (2000). Fear-avoidance and its consequences in chronic musculoskeletal pain: a state of the art. Pain.

[25] Sullivan M, Bishop S, Pivik J (1995). The pain catastrophizing scale: development and validation. Psychological Assessment.

[26] Vowles KE, McCracken LM, Eccleston C (2007). Processes of change in treatment for chronic pain: the contributions of pain, acceptance, and catastrophizing. Eur J Pain.

[27] Arnow BA, Blasey CM, Constantino MJ, Robinson R, Hunkeler E, Lee J, Fireman B, Khaylis A, Feiner L, Hayward C (2011). Catastrophizing, depression and pain-related disability. Gen Hosp Psychiatry.

[28] McCracken LM (2005). Contextual Cognitive-Behavioral Therapy for Chronic Pain.

[29] Hayes S, Strosahl K, Wilson K (2003). Acceptance and Commitment Therapy: An Experiential Approach to Behavior Change.

[30] McCracken LM, McCracken LM (2011). Mindfulness and Acceptance in Behavioral Medicine: Current Theory and Practice (The Context Press Mindfulness and Acceptance Practica Series).

[31] Wetherell JL, Afari N, Rutledge T, Sorrell JT, Stoddard JA, Petkus AJ, Solomon BC, Lehman DH, Liu L, Lang AJ, Atkinson JH (2011). A randomized, controlled trial of acceptance and commitment therapy and cognitive-behavioral therapy for chronic pain. Pain.

[32] Vowles KE, Thompson M, McCracken LM (2011). Acceptance and Commitment Therapy for chronic pain. Mindfulness and Acceptance in Behavioral Medicine: Current Theory and Practice (The Context Press Mindfulness and Acceptance Practica Series).

[33] Bailey KM, Carleton RN, Vlaeyen JW, Asmundson GJ (2010). Treatments addressing pain-related fear and anxiety in patients with chronic musculoskeletal pain: a preliminary review. Cogn Behav Ther.

[34] Dahl J, Luciano C, Hayes SC, Wilson, KG (2005). Acceptance and Commitment Therapy for Chronic Pain.

[35] Ryan RM, Deci EL (2000). Self-determination theory and the facilitation of intrinsic motivation, social development, and well-being. Am Psychol.

[36] Petty RE, Cacioppo JT (1986). The elaboration likelihood model of persuasion. Adv Exp Soc Psychol.

[37] Cassidy EL, Atherton RJ, Robertson N, Walsh DA, Gillett R (2012). Mindfulness, functioning and catastrophizing after multidisciplinary pain management for chronic low back pain. Pain.

[38] McCracken LM, Vowles KE, Eccleston C (2004). Acceptance of chronic pain: component analysis and a revised assessment method. Pain.

[39] Goldberg DP, Gater R, Sartorius N, Ustun TB, Piccinelli M, Gureje O, Rutter C (1997). The validity of two versions of the GHQ in the WHO study of mental illness in general health care. Psychol Med.

[40] McCracken LM, Yang SY (2006). The role of values in a contextual cognitive-behavioral approach to chronic pain. Pain.

[41] Burckhardt CS, Clark SR, Bennett RM (1991). The fibromyalgia impact questionnaire: development and validation. J Rheumatol.

[42] Saris-Baglama RN, Dewey CJ, Chisholm GB, Plumb E, King J, Rasicot P, Kosinski M, Bjorner JB, Ware JE (2011). QualityMetric Outcomes Scoring Software 4.5. User's Guide.

[43] Simons DG, Travell JG, Simons LS (1999). Travell & Simons' Myofascial Pain and Dysfunction: The Trigger Point Manual Volume 1.

[44] Simons DG, Travell (1992). Travell & Simons' Myofascial Pain and Dysfunction: The Trigger Point Manual Volume 2.

[45] Scollon C, Kim-Prieto C, Diener E (2003). Experience Sampling: Promises and Pitfalls, Strengths and Weaknesses. Journal of Happiness Studies.

[46] Van Damme S, Crombez G, Bijttebier P, Goubert L, Van Houdenhove B (2002). A confirmatory factor analysis of the Pain Catastrophizing Scale: invariant factor structure across clinical and non-clinical populations. Pain.

[47] Severeijns R, Vlaeyen JW, van den Hout MA, Picavet HS (2004). Pain catastrophizing is associated with health indices in musculoskeletal pain: a cross-sectional study in the Dutch community. Health Psychol.

[48] Andersson G (2009). Using the Internet to provide cognitive behaviour therapy. Behav Res Ther.

[49] Cohen J (1988). Statistical Power Analysis for the Behavioral Sciences.

[50] Proudfoot J, Klein B, Barak A, Carlbring P, Cuijpers P, Lange A, Ritterband L, Andersson G (2011). Establishing guidelines for executing and reporting internet intervention research. Cognitive Behaviour Therapy.

[51] Hayes SC, Luoma JB, Bond FW, Masuda A, Lillis J (2006). Acceptance and commitment therapy: model, processes and outcomes. Behav Res Ther.

[52] Winterowd C, Beck AT, Gruener D (2003). Cognitive Therapy with Chronic Pain Patients.

[53] Schütze R, Rees C, Preece M, Schütze M (2010). Low mindfulness predicts pain catastrophizing in a fear-avoidance model of chronic pain. Pain.

[54] Vowles KE, McCracken LM, Eccleston C (2008). Patient functioning and catastrophizing in chronic pain: the mediating effects of acceptance. Health Psychol.

[55] Jensen KB, Kosek E, Wicksell R, Kemani M, Olsson G, Merle JV, Kadetoff D, Ingvar M (2012). Cognitive Behavioral Therapy increases pain-evoked activation of the prefrontal cortex in patients with fibromyalgia. Pain.

[56] Veehof MM, Oskam MJ, Schreurs KM, Bohlmeijer ET (2011). Acceptance-based interventions for the treatment of chronic pain: a systematic review and meta-analysis. Pain.

[57] McCracken LM, Williams JL, Tang NK (2011). Psychological flexibility may reduce insomnia in persons with chronic pain: a preliminary retrospective study. Pain Med.

[58] Johnston M, Foster M, Shennan J, Starkey NJ, Johnson A (2010). The effectiveness of an Acceptance and Commitment Therapy self-help intervention for chronic pain. Clin J Pain.

[59] Thorsell J, Finnes A, Dahl J, Lundgren T, Gybrant M, Gordh T, Buhrman M (2011). A comparative study of 2 manual-based self-help interventions, acceptance and commitment therapy and applied relaxation, for persons with chronic pain. Clin J Pain.

[60] Fjeldsoe BS, Marshall AL, Miller YD (2009). Behavior change interventions delivered by mobile telephone short-message service. Am J Prev Med.

[61] Wei J, Hollin I, Kachnowski S (2011). A review of the use of mobile phone text messaging in clinical and healthy behaviour interventions. J Telemed Telecare.

[62] Cuijpers P, van Straten A, Andersson G (2008). Internet-administered cognitive behavior therapy for health problems: a systematic review. J Behav Med.

[63] Jelin E, Granum V, Eide H (2012). Experiences of a web-based nursing intervention--interviews with women with chronic musculoskeletal pain. Pain Manag Nurs.

[64] Streiner DL (2008). Missing data and the trouble with LOCF. Evid Based Ment Health.

[65] Karjalainen K, Malmivaara A, van Tulder M, Roine R, Jauhiainen M, Hurri H, Koes B (2000). Multidisciplinary rehabilitation for fibromyalgia and musculoskeletal pain in working age adults. Cochrane Database Syst Rev.

[66] Eysenbach G, CONSORT-EHEALTH Group (2011). CONSORT-EHEALTH: Improving and Standardizing Evaluation Reports of Web-based and Mobile Health Interventions. J Med Internet Res.

