# Supplementary material for: A Smartphone-Based Intervention With Diaries and Therapist-Feedback to Reduce Catastrophizing and Increase Functioning in Women With Chronic Widespread Pain: Randomized Controlled Trial
Source: J Med Internet Res. 2013 Jan 7;15(1):e5. doi: 10.2196/jmir.2249 (PMC3636250; doi:10.2196/jmir.2249)
Supplement: Supplementary file 1 [file jmir_v15i1e5_app1.pdf]

CONSORT-EHEALTH Checklist V1.6 Report

**Manuscript Number**	2249
**Date completed**	8/25/2011 14:50:29
**by**	Olöf Birna Kristjansdottir

“A smartphone-based intervention with diaries and therapist-feedback to reduce catastrophizing and increase functioning in women with chronic widespread pain”.

TITLE

1a-i) Identify the mode of delivery in the title

Yes. “A smartphone-based intervention with diaries and therapist-feedback to reduce catastrophizing and increase functioning in women with chronic widespread pain”.

1a-ii) Non-web-based components or important co-interventions in title

Yes. “A smartphone-based intervention with diaries and therapist-feedback to reduce catastrophizing and increase functioning in women with chronic widespread pain”.

1a-iii) Primary condition or target group in the title

Yes. “A smartphone-based intervention with diaries and therapist-feedback to reduce catastrophizing and increase functioning in women with chronic widespread pain”.

ABSTRACT

1b-i) Key features/functionalities/components of the intervention and comparator in the METHODS section of the ABSTRACT

Yes. “One hundred forty and women with chronic widespread pain, who participated in a 4-week inpatient rehabilitation program, were randomized into two groups: With or without a smartphone intervention after the rehabilitation. The smartphone intervention consisted of one face-to-face session and 4 weeks of written communication via a smartphone. Participants received three smartphone diary entries daily to support their self-monitoring of and reflection on pain-related thoughts, feelings and activities. The registered diaries were immediately available to a therapist, who submitted tailored written feedback daily based on cognitive behavioral principles. Both groups were given access to a non-interactive Web site after discharge to promote constructive self-management.”

1b-ii) Level of human involvement in the METHODS section of the ABSTRACT

Yes. “The registered diaries were immediately available to a therapist, who submitted tailored written feedback daily based on cognitive behavioral principles.”

1b-iii) Open vs. closed, web-based (self-assessment) vs. face-to-face assessments in the METHODS section of the ABSTRACT

Yes. “One hundred forty and women with chronic widespread pain, who participated in a 4-week inpatient rehabilitation program, were randomized into two groups: With or without a smartphone intervention after the rehabilitation. The smartphone intervention consisted of one face-to-face session and 4 weeks of written communication via a smartphone.”

1b-iv) RESULTS section in abstract must contain use data

Yes. “One hundred and twelve women completed the study: 48 in the intervention group and 64 in the control group. Immediately after the intervention period, the intervention group reported less catastrophizing (M=9.20, SD=5.85) than the control group (M=15.71, SD=9.11, P<0.001), yielding a large effect size (Cohen’s d=0.87) for study completers. At 5-month follow-up, the between-group effect sizes remained moderate for catastrophizing (0.74, P=0.003), acceptance of pain (0.54, P=0.02) and functioning and symptom levels (0.75, P=0.001).”

1b-v) CONCLUSIONS/DISCUSSION in abstract for negative trials

Yes. “The results suggest that a smartphone-delivered intervention with diaries and situational feedback can reduce catastrophizing and prevent increase in functional impairment and symptom levels in women with chronic widespread pain following inpatient rehabilitation.”

INTRODUCTION

2a-i) Problem and the type of system/solution

Yes. “Multidimensional rehabilitation, including physical exercise and cognitive behavioral therapy (CBT), is recommended as treatment [6-7]. A key element is self-management, e.g. balancing activity and rest, stress management and emotion regulation, and doing appropriate physical exercises [9]. However, relapse of symptoms is not uncommon [6, 7, 10, 11] since self-management can be challenging due to the nature of the symptoms. Few studies have examined home-delivered interventions that aim to support self-management of chronic pain following rehabilitation [11, 12] “

2a-ii) Scientific background, rationale: What is known about the (type of) system

Yes. "Internet-based interventions using cognitive behavioral approaches can be effective in promoting self-management of chronic pain conditions [13-15]. Web-based programs delivered via smartphones are increasingly used to support the self-management of various health disorders; however research on smartphone interventions for patients with chronic pain is limited [16]. Among the advantages of using smartphones rather than the traditional personal computer are their small size and mobility, making self-management support available to the user in most situations [16]. Diaries with questions intended to support self-monitoring and reflection are made available on the phone and the registered information can be submitted to a Web site and made instantly available to a therapist. Feedback can be automatically delivered and, to some extent, tailored to the registered information, or it can be even more individualized by a therapist [17-19]."

METHODS

3a) CONSORT

Yes. "The present randomized, controlled trial investigates the efficacy of a smartphone intervention on catastrophizing, acceptance, emotional distress, values-based behavior and functioning and symptom level in women with CWP who had completed a 4-week inpatient rehabilitation program. For the first 4 weeks after discharge, the intervention group received a Web-based intervention comprising the self-monitoring of symptoms, thoughts, feelings and self-management behavior through daily smartphone diaries and written tailored CBT-based feedback. It was hypothesized that the intervention group would show less catastrophizing and emotional distress, more acceptance of pain and success in values-based living, and improved functioning and symptom levels after completing the intervention period and at 5-month follow-up, compared to the control group."

3b-i) Bug fixes, Downtimes, Content Changes

No content changes were made. No major downtime was experienced.

4a-i) Computer / Internet literacy

Yes. "being able to use a smartphone".

4a-ii) Open vs. closed, web-based vs. face-to-face assessments:

Yes. "Participants were recruited consecutively from Jeløy Kurbad Rehabilitation Centre in Moss, Norway. Patients were referred to the center by their general practitioner or a medical specialist."

4a-iii) Information giving during recruitment

Yes. "At admission to the inpatient rehabilitation program, all CWP patients received a written invitation to attend an informational group meeting at which a researcher or a research assistant presented the study. Those who were interested in participating and met the inclusion criteria were given an informed consent form to sign. "

4b-i) Report if outcomes were (self-)assessed through online questionnaires

Online questionnaires not used.

4b-ii) Report how institutional affiliations are displayed

5-i) Mention names, credential, affiliations of the developers, sponsors, and owners

"The study is funded by the Research Council of Norway (grant number 182014). "

To software developers, Erlend Eide og Mads Lien, were affilated to the Oslo and Akershus University Collage of Applied Sciences.

5-ii) Describe the history/development process

Yes. "The intervention was developed in 2008. One of the authors (EE) was responsible for the software development. The usability of the intervention was tested in a pretrial study with six women with chronic pain."

5-iii) Revisions and updating

The website for all participants was static and no changes was made to it during the study period.

5-iv) Quality assurance methods

The material on the website was quality assured by the project leader, dr. Hilde Eide. The written feedback from therapist was supervised: "Two members of the group supervised the content of the feedback. They had extensive experience in teaching mindfulness meditation (HE) and supervising CBT/ACT (EAF)."

5-v) Ensure replicability by publishing the source code, and/or providing screenshots/screen-capture video, and/or providing flowcharts of the algorithms used

Screen shot is provided to provide example of the smartphone scren/diaries.

5-vi) Digital preservation

We have not provided URIL to the website, since it is no longer running.

5-vii) Access

Smartphone-intervention:
"The participant was lent a smartphone (HTC TyTN II) with a touch screen and a keyboard." The diaires and feedback was accecable only from the smartphone.
Web site:
"All participants received access to a static Web site with information on self-management strategies for people with chronic pain; not anticipated to have large effect on the study outcomes on its own."- Participants received a password to use for log-in.

5-viii) Mode of delivery, features/functionalities/components of the intervention and comparator, and the theoretical framework

Mode of delivery:
Smartphone intervention: Real time self-report on questions in diaries. Written communication from therapist with maximum 1 hour delay from the real time diary.

Components/Description of the content: :
"The smartphone intervention had the following four components:
(1) Face to face session. The intervention started with a 1-hour individual session between a nurse working on the project and the participant. The session took place in the last week before discharge. Each participant was informed about the intervention and asked about functioning, goals for health-related behavior and support needs. Values and values-based activities were discussed and the patient received two written values-based exercises to take home. The participant was lent a smartphone (HTC TyTN II) with a touch screen and a keyboard. The participants received information (name and qualifications) about their therapist for the intervention, which, in some cases was the nurse at the meeting. The nurse attending the face-to-face session summarized the meeting and sent it to the relevant therapist.
(2) Web-based diaries. The participant was asked to complete three diary entries per day using the smartphone.
The diaries included 16 - 24 questions about the current level and interference of pain, feelings and thoughts related to avoidance, catastrophizing and acceptance. They also included questions about planned and previous use of self-management activities and daily values-based and practical activities. Lists of self-management activities (e.g. mild exercise, stretching, resting, aerobic exercise, pleasurable activity) were provided as a reminder. The questions were chosen to support self-monitoring and reflection and were formulated in accordance with the Experience Sampling Method principles designed to capture experience in real time without retrospective bias (e.g. “Right now I am feeling…”) [43]. See Table 1 for examples of the questions. Participants answered most questions by choosing predefined alternatives or scoring five-point Likert scales. All diaries included a comment field giving participants the opportunity to write a short personal message to the therapist.
The morning and evening diary entries were sent at fixed hours chosen by each participant. The second diary entry of the day was sent at a time randomly chosen by the Web server, between 11 am and 2 pm. The purpose of including three diary entries, including one at a randomly chosen time, was to encourage self-monitoring and reflection at different hours and in different situations. At the time scheduled for diary completion, a Short Message Service (SMS) message with a link to a secure Web site, where the diary could be opened and questions answered and posted, was received by the participant. The participants completed the first diary entry during the face-to-face session, and continued during the last week before discharge with the goal of getting used to the diaries before discharge (a run-in period). After discharge the diaries were received for 4 weeks. The participant could call a member of the research group (OBK, HE) for technical support. No data were kept on the mobile phone. Two automated SMS reminders were sent, if needed, within 1 hour of the first signal.
(3) Written situational feedback. For 4 weeks after discharge, excluding weekends, participants received daily written feedback from a therapist on the information they had given in their diaries. The feedback was tailored to each participant’s situation as reported in the diary. It was written in an empathic style and included repetition of content reported in the diaries, positive reinforcement, reminders of self-management information given at the rehabilitation center, ACT exercises and reflective questions. The aim was to encourage nonjudgmental awareness of catastrophizing and to stimulate mindfulness and willingness to engage in meaningful activities despite pain or other discouraging intrusions (Table 1). The instructions for the exercises were written directly in the feedback or the participant was referred to exercises available on the mobile phone and/or the Web site, see below. The feedback was also tailored to the summary of personal information given at the face-to-face session (e.g. family situation and health-related goals) and results on self-reported discrepancy between values and values-based living assessed with the CPVI at the end of the rehabilitation program. The feedback was usually available for the participant within 90 minutes of completing the second diary of the day. If this diary was not submitted feedback based on information from the latest submitted diary was sent. When the feedback was available, the participant received an SMS with a link to the Web site where the feedback could be found. There was no limitation on the length of the feedback, which ranged from a few sentences to a few paragraphs.
The feedback was written by any of three of the authors (OBK, TLS and HE); each participant received signed feedback from the same person throughout the intervention. All therapists had a background in health care sciences (nursing and/or psychology) and had received training in ACT. The feedback protocol was based on ACT for chronic pain [21, 30] with a different focus during each of the 4 weeks. For example, in the first week the focus was on supporting the participant to continue doing the exercises/stretches as recommended at the inpatient program, and during the second week, simple mindfulness exercises were introduced (e.g. a few minutes of focused breathing). Once a week, the feedback included an invitation to a values reflection exercise, and every week, questions were included to stimulate reflection on health-related goals. The last feedback comprised a written summary of the registered diary information during the 4-week period. Content from the growing “bank” of feedback written by all the therapists was used for other participants when appropriate according to the registered information. It took 10-15 minutes, on average, to write each piece of feedback. Two members of the group supervised the content of the feedback. They had extensive experience in teaching mindfulness meditation (HE) and supervising CBT/ACT (EAF)."

Controlgroup:
"All participants received access to a static Web site with information on self-management strategies for people with chronic pain; not anticipated to have large effect on the study outcomes on its own." Participants received a password to use for log in.

have large effect on the study outcomes on its own." - Participants received a password to use for log-in.

Theoretical framework:
"Cognitive and emotional factors influence the pain experience [22]. Among the psychological constructs that can play an important role in the development and maintenance of chronic pain is catastrophizing [23, 24]. Pain-related catastrophizing includes the tendency to ruminate about and magnify symptoms, to expect the worst and to feel helpless regarding self-management [25]. Catastrophizing tends to discourage patients from committing to their valued behavior and has consistently been found to predict distress and disability [24, 25]. In rehabilitation, catastrophizing is targeted in a number of ways, such as with CBT and exercise programs [26]. However, interventions delivered in the patient's private environment, supporting self-monitoring and providing tailored feedback, may further help reducing catastrophizing [10, 12]. A mobile phone-delivered intervention with diaries and daily CBT-based feedback has been found to reduce catastrophizing thoughts in patients with irritable bowel syndrome and the effects were maintained at a 3-month follow-up [17].
Acceptance and Commitment Therapy (ACT) is a third-generation CBT based on the notion that suffering may largely be caused by our thinking about painful experiences rather than the experiences themselves [27]. Suffering can be reduced through mindfulness, acceptance and committed action [27]. ACT has been found effective for people with various chronic health disorders [27]; and has been used successfully to reduce catastrophizing and disability in chronic pain patients [30-32]. The goals are to promote psychological flexibility, i.e. acceptance of, rather than struggle with, unwanted thoughts, emotions and symptoms (e.g. pain or catastrophizing) and to increase commitment to personal values [26, 27]. A person's values are described as his or hers desired way of being within various life domains (e.g. being a caring friend). Values differ from goals in that they can never be fully obtained, but can give a continuous sense of motivation, direction and purpose [32]. The focus on values is also evident in the Self-Determination Theory (SDT); which states the importance of perceiving behavior as self-determined for intrinsic motivation to be maintained [33]. According to the SDT, context specific feedback can play a role in enhancing intrinsic motivation to maintain behavior [33]. Guidance was also found in the persuasion theory "Elaboration Likelihood Model" [34]. This theory specifies how information can be constructed and presented to enhance either cognitive elaboration or emotional elaboration intending to influence behavior change. Elements focused on in this study are repetition, personal relevance and involvement influencing the cognitive level, and influencing emotional pathways through emotion recognition, mindfulness exercises and empathic communication."

Control group:
"All participants received access to a static Web site with information on self-management strategies for people with chronic pain; not anticipated to have large effect on the study outcomes on its own. The Web site included a few written ACT exercises and audio files with mindfulness exercises (as described above). An example of the written exercises is a behavior analysis aiming to strengthen the ability to observe thought content, feelings and behavior and the connection between these (adapted from [26])."

5-ix) Describe use parameters

The control group got the instructions to use the website as they suited.

The smartphone intervention:
"The participant was asked to complete three diary entries per day using the smartphone."

"For 4 weeks after discharge, excluding weekends, participants received daily written feedback from a therapist on the information they had given in their diaries. The feedback was tailored to each participant's situation as reported in the diary. It was written in an empathic style and included repetition of content reported in the diaries, positive reinforcement, reminders of self-management information given at the rehabilitation center, ACT exercises and reflective questions. The aim was to encourage nonjudgmental awareness of catastrophizing and to stimulate mindfulness and willingness to engage in meaningful activities despite pain or other discouraging intrusions (Table 1). The instructions for the exercises were written directly in the feedback or the participant was referred to exercises available on the mobile phone and/or the Web site."

5-x) Clarify the level of human involvement

"(1) Face to face session. The intervention started with a 1-hour individual session between a nurse working on the project and the participant. The session took place in the last week before discharge. Each participant was informed about the intervention and asked about functioning, goals for health-related behavior and support needs. Values and values-based activities were discussed and the patient received two written values-based exercises to take home. The participant was lent a smartphone (HTC TyTN II) with a touch screen and a keyboard. The participants received information (name and qualifications) about their therapist for the intervention, which, in some cases was the nurse at the meeting. The nurse attending the face-to-face session summarized the meeting and sent it to the relevant therapist."
"For 4 weeks after discharge, excluding weekends, participants received daily written feedback from a therapist on the information they had given in their diaries."

5-xi) Report any prompts/reminders used

As reminders to fill out the diaries: "Two automated SMS reminders were sent, if needed, within 1 hour of the first signal."

5-xii) Describe any co-interventions (incl. training/support)
"Face to face session. The intervention started with a 1-hour individual session between a nurse working on the project and the participant. The session took place in the last week before discharge. Each participant was informed about the intervention and asked about functioning, goals for health-related behavior and support needs. Values and values-based activities were discussed and the patient received two written values-based exercises to take home. The participant was lent a smartphone (HTC TyTN II) with a touch screen and a keyboard. The participants received information (name and qualifications) about their therapist for the intervention, which, in some cases was the nurse at the meeting. The nurse attending the face-to-face session summarized the meeting and sent it to the relevant therapist."
6a-i) Online questionnaires: describe if they were validated for online use and apply CHERRIES items to describe how the questionnaires were designed/deployed
Online questionnaires were not used.
6a-ii) Describe whether and how "use" (including intensity of use/dosage) was defined/measured/monitored
"Use of the non-interactive Web site was assessed with a self-report four weeks after discharge (T3) on how often the participant had visited the Web site."

"The response rate to the diary entries during the 4 weeks after discharge ranged from 27.4 – 95.2%, with a mean of 68.5% and a median of 70.2%. Most (83%) participants received 84 entries (4 weeks). 17% received additional days of entries to compensate for holidays to ensure 20 days with registration and feedback."
6a-iii) Describe whether, how, and when qualitative feedback from participants was obtained
Evaluation questionnaires were mailed to all participants after the intervention period but are not reported on here.
18 of the participants were interviewed by two M.Sc. students in nursing to evaluate their experience of the intervention. One of them has published the study: "In a qualitative study with seven of our participants the intervention was described as consciousness expanding, motivating and supportive [62]."
7a-i) Describe whether and how expected attrition was taken into account when calculating the sample size
"Power analyses were based on the level of reported catastrophizing in CWP samples [5, 19, 44,45], a moderate effect size (Cohen's d=0.5) and allowing for attrition commonly seen in studies on Internet interventions [46]. A sample size of 70 participants per group was needed to detect a moderate effect size in the primary outcome variable with a two-sided 5% significance level and 80% power. "
7b) CONSORT
Not relevant.
8a) CONSORT
"A computer-generated sequence list with the two groups randomized in blocks of four was used. A research assistant put the allocation information in sequentially numbered envelopes and sealed them. A researcher subsequently gave each participant a number and opened the matched envelope to reveal the group allocation. The information about group allocation was revealed to the participant at a meeting with a nurse in the last week of the inpatient program."
8b) CONSORT
"The design is a parallel-group, randomized controlled trial. Block randomization was used for practical reasons to ensure similar numbers in each group at each time point."
9) CONSORT
""A computer-generated sequence list with the two groups randomized in blocks of four was used. A research assistant put the allocation information in sequentially numbered envelopes and sealed them. A researcher subsequently gave each participant a number and opened the matched envelope to reveal the group allocation. The information about group allocation was revealed to the participant at a meeting with a nurse in the last week of the inpatient program.""
10) CONSORT
""A computer-generated sequence list with the two groups randomized in blocks of four was used. A research assistant put the allocation information in sequentially numbered envelopes and sealed them. A researcher subsequently gave each participant a number and opened the matched envelope to reveal the group allocation. The information about group allocation was revealed to the participant at a meeting with a nurse in the last week of the inpatient program."
11a-i) Specify who was blinded, and who wasn't
Neither participants or care providers were blinded. Those doing data analysis were not blinded.
11a-ii) Discuss e.g., whether participants knew which intervention was the "intervention of interest" and which one was the "comparator"
It was suggested in the way the information was presented that the smartphone-intervention was intervention of interest.
11b) CONSORT
Not relevant.

12a) CONSORT
"To investigate differences in demographic variables and baseline characteristics, independent sample t-tests and chi-square tests were used. Data was checked for normality distribution and when found suitable for parametric analyses, t-tests were used, and otherwise non-parametric tests (Mann-Whitney) were applied. The Cohen's d effect sizes were calculated using the difference between the groups' means divided by the mean standard deviation of both groups. If one or two items were missing on the GHQ, they were scored as the absence of the symptom (0). If another instrument included one or two missing items, the item/-s were replaced with the mean of other items from the participant's instrument. If two response alternatives were marked, the healthier option was chosen. Total score was not computed if more than two items were missing, and the case was categorized as missing a total score for the instrument. The number of participants included in each analysis is given. In the intention-to-treat analysis the last observed value was carried forward where data was missing. Five of the participants who withdrew from the smartphone intervention sent in questionnaires at T3 and seven at the 5-month follow-up (T4). The intention-to-treat analysis included all participants except those who met the exclusion criteria after randomization (n=135). In the analysis of secondary outcomes, only those who completed the interventions were included (n=112). A significance level of P=0.05 was used and a tendency toward difference was defined as $p < 0.1$. Effect sizes were categorized as small (<0.5), medium (0.5 - 0.8) and large (>0.8) in accordance with Cohen [47]."
12a-i) Imputation techniques to deal with attrition / missing values
"The intention-to-treat analysis included all participants except those who met the exclusion criteria after randomization (n=135). In the analysis of secondary outcomes, only those who completed the interventions were included (n=112)."
12b) CONSORT
"In the analysis of secondary outcomes, only those who completed the interventions were included (n=112)."

RESULTS

13a) CONSORT
Table 3.
13b) CONSORT
Included in Figure 1.
13b-i) Attrition diagram
Figure 1.
14a) CONSORT
"Participants completed self-administered questionnaires on arrival at the rehabilitation center (T1), at discharge (T2), immediately after the smartphone intervention (T3), and 6 months after discharge from the rehabilitation center (T4), which was 5 months after the smartphone intervention and 5 months after smartphone completion (i.e. 6 months after discharge from the rehabilitation center). "
14a-i) Indicate if critical "secular events" fell into the study period
Not relevant.
14b) CONSORT
Not relevant.
15) CONSORT
Table 2.
15-i) Report demographics associated with digital divide issues
Table 2.
16-i) Report multiple "denominators" and provide definitions
"The intention-to-treat analysis included all participants except those who met the exclusion criteria after randomization (n=135). In the analysis of secondary outcomes, only those who completed the interventions were included (n=112)."
Per-protocol analysis also stated in tables.
16-ii) Primary analysis should be intent-to-treat
"The intention-to-treat analysis included all participants except those who met the exclusion criteria after randomization (n=135).
Primary analysis was intent-to-treat.
17a) CONSORT
Provided in Table 7.
17a-i) Presentation of process outcomes such as metrics of use and intensity of use

"Response rate to the smartphone diary entries
The response rate to the diary entries during the 4 weeks after discharge ranged from 27.4 – 95.2%, with a mean of 68.5% and a median of 70.2%. Most (83%) participants received 84 entries (4 weeks). 17% received additional days of entries to compensate for holidays to ensure 20 days with registration and feedback.

Use of the non-interactive Web site:
Of the completers in the smartphone intervention, 22 (45.8%) reported never visiting the Web site. Six (12.5%) visited it once; eight women (16.7%) viewed it twice, and 11 (22.9%) three times or more. One did not respond to the question. Thirty-eight completers in the control group answered the question. Twelve (18.8%) reported never having visited the Web site, five (7.8%) had viewed it once, nine (14.1%) twice and twelve (18.8%) had visited three or more times."

17b) CONSORT

Not relevant.

18) CONSORT

Not relevant.

18-i) Subgroup analysis of comparing only users

"The present study has some limitations. The generalizability of the results is reduced by several factors. Firstly, the intervention group had a withdrawal rate of 30% and this might have resulted in differences in the characteristics of completers between groups. Indeed, there was a trend towards the completers being younger and having less pain, fatigue and better function measured with SF-8 at baseline"

19) CONSORT

Not applicable.

19-i) Include privacy breaches, technical problems

A temporary problem with submitting the diary forms was reported occationally.

19-ii) Include qualitative feedback from participants or observations from staff/researchers

This is published elsewhere.

DISCUSSION

20-i) Typical limitations in ehealth trials

"The present study has some limitations. The generalizability of the results is reduced by several factors. Firstly, the intervention group had a withdrawal rate of 30% and this might have resulted in differences in the characteristics of completers between groups. Indeed, there was a trend towards the completers being younger and having less pain, fatigue and better function measured with SF-8 at baseline. Our intention with a run-in period during the last week of the inpatient program was to give the patients a chance to get used to the smartphone diaries before returning home. Our results may indicate that this might not have been suitable for all participants; it might have been more feasible to give the participants the choice of starting the intervention after discharge from the inpatient program. During the inpatient program the participants had a busy schedule with activities and may therefore have experienced adding the smartphone diaries as stressful. They chose to receive their morning and evening diaries at hours suitable for their schedule at home, which may possibly have been inconvenient while still at the rehabilitation center. No differences in dropout rates were found between employment statuses. High withdrawal rates have been a challenge in SMS-based and Web-based interventions [59]. In a review of 17 trials of Internet self-management interventions for people with chronic pain the withdrawal rate ranged from 6% to 59% with a median withdrawal rate of 27% [13]. Therapist contact and tailored messages have been found to correlate with lower withdrawal rates, but as our results shows, other factors clearly also play roles. Despite the considerable withdrawal rate, many experienced the present intervention positively. In a qualitative study with seven of our participants the intervention was described as consciousness expanding, motivating and supportive [62]. Secondly, the response rate to assessment questionnaires was below 70% at both follow-ups; this affects the generalizability of the results because data cannot be assumed to be missing at random. The response rate was different between the groups, with a lower response rate in the control group immediately after the intervention period. This is commonly experienced in randomized controlled studies [63]. Those who did not return questionnaires after the intervention period (T3) had lower pain level at baseline (T1) than those who did. Also, those who did not return questionnaire after the smartphone intervention period (T3) had more function and symptom impairment at discharge from the center compared with those who returned those questionnaires. Since all except one in the smartphone group returned the questionnaires after the intervention (T3) and those not responding belonged to the control group, it may be that the level of functional impairment and symptom severity for the control group was, in fact, higher. The 5-month follow-up results could also be affected since there was a trend toward those not returning the questionnaires reporting less pain at baseline (T1) and better functioning and less symptom severity at discharge (T2). At last, the generalizability is also affected by the fact that just over half of those eligible to participate were included in the study. We do not know if those who choose to participate differed in any way from those who declined participation. The introduction meeting for the study was scheduled during the second week of the rehabilitation program. For some it may have been too early to consider involvement in a follow-up intervention and others may have used the opportunity to prioritize private time, in the tight rehabilitation schedule, instead of listening to study information. Moreover, in the stress management part of the rehabilitation program, the patients were encouraged to set limits and say no to requests they felt added more stress to their everyday burden. Patients with high self-efficacy regarding coping after discharge may have been more likely to not attend the informational meeting. Also, since all eligible to the study received a short information letter about study, some may have found the intervention format unsuitable. The increase in function impairment and symptom levels in the control group after discharge is not in line with the results of a study on 200 patients with CWP or fibromyalgia participating in the same kind of 4-week inpatient program at the same rehabilitation center. The results of the study showed significant improvements in functioning and symptom levels, maintained at both 6- and 12- month follow-ups [8]."

21-i) Generalizability to other populations

"The present study has some limitations. The generalizability of the results is reduced by several factors. Firstly, the intervention group had a withdrawal rate of 30% and this might have resulted in differences in the characteristics of completers between groups. Indeed, there was a trend towards the completers being younger and having less pain, fatigue and better function measured with SF-8 at baseline. Our intention with a run-in period during the last week of the inpatient program was to give the patients a chance to get used to the smartphone diaries before returning home. Our results may indicate that this might not have been suitable for all participants; it might have been more feasible to give the participants the choice of starting the intervention after discharge from the inpatient program. During the inpatient program the participants had a busy schedule with activities and may therefore have experienced adding the smartphone diaries as stressful. They chose to receive their morning and evening diaries at hours suitable for their schedule at home, which may possibly have been inconvenient while still at the rehabilitation center. No differences in dropout rates were found between employment statuses. High withdrawal rates have been a challenge in SMS-based and Web-based interventions [59]. In a review of 17 trials of Internet self-management interventions for people with chronic pain the withdrawal rate ranged from 6% to 59% with a median withdrawal rate of 27% [13]. Therapist contact and tailored messages have been found to correlate with lower withdrawal rates, but as our results shows, other factors clearly also play roles. Despite the considerable withdrawal rate, many experienced the present intervention positively. In a qualitative study with seven of our participants the intervention was described as consciousness expanding, motivating and supportive [62]. Secondly, the response rate to assessment questionnaires was below 70% at both follow-ups; this affects the generalizability of the results because data cannot be assumed to be missing at random. The response rate was different between the groups, with a lower response rate in the control group immediately after the intervention period. This is commonly experienced in randomized controlled studies [63]. Those who did not return questionnaires after the intervention period (T3) had lower pain level at baseline (T1) than those who did. Also, those who did not return questionnaire after the smartphone intervention period (T3) had more function and symptom impairment at discharge from the center compared with those who returned those questionnaires. Since all except one in the smartphone group returned the questionnaires after the intervention (T3) and those not responding belonged to the control group, it may be that the level of functional impairment and symptom severity for the control group was, in fact, higher. The 5-month follow-up results could also be affected since there was a trend toward those not returning the questionnaires reporting less pain at baseline (T1) and better functioning and less symptom severity at discharge (T2). At last, the generalizability is also affected by the fact that just over half of those eligible to participate were included in the study. We do not know if those who choose to participate differed in any way from those who declined participation. The introduction meeting for the study was scheduled during the second week of the rehabilitation program. For some it may have been too early to consider involvement in a follow-up intervention and others may have used the opportunity to prioritize private time, in the tight rehabilitation schedule, instead of listening to study information. Moreover, in the stress management part of the rehabilitation program, the patients were encouraged to set limits and say no to requests they felt added more stress to their everyday burden. Patients with high self-efficacy regarding coping after discharge may have been more likely to not attend the informational meeting. Also, since all eligible to the study received a short information letter about study, some may have found the intervention format unsuitable. The increase in function impairment and symptom levels in the control group after discharge is not in line with the results of a study on 200 patients with CWP or fibromyalgia participating in the same kind of 4-week inpatient program at the same rehabilitation center. The results of the study showed significant improvements in functioning and symptom levels, maintained at both 6- and 12- month follow-ups [8]."

21-ii) Discuss if there were elements in the RCT that would be different in a routine application setting

In a routine application setting, the nurse/therapist would perhaps be employed at the rehabilitation center.

22-i) Restate study questions and summarize the answers suggested by the data, starting with primary outcomes and process outcomes (use)

"The results from the per-protocol analysis indicate that this intervention with diaries and written tailored feedback reduced catastrophizing and increased acceptance in women with CWP and that the effects persisted 5 months after the end of the intervention. At a 5-month follow-up the control group experienced increased emotional distress compared to the distress at discharge from the inpatient program, whereas the smartphone group did not. The between group effect size on functioning and symptom level was close to large (0.75) at the 5-month follow-up measured with the FIQ, but no difference was seen in the physical component of the SF-8."

22-ii) Highlight unanswered new questions, suggest future research

"Future research could investigate whether automatic feedback could be effectively tailored to diaries and integrated in an application to reduce the investment of human resource used in the presented intervention.

...Our results give preliminary support to the efficacy of a smartphone-intervention on catastrophizing, functioning and symptom level in women with chronic widespread pain. In addition to subgroup analyses of participants and results on long-term effects, research on practice implications, innovation and added values for the users are needed."

Other information

23) CONSORT

Trial Registration: NCT01236209; http://clinicaltrial.gov/ct2/results?term=nct01236209

24) CONSORT

The full trial protocol has not been published.

25) CONSORT

"The study is funded by the Research Council of Norway (grant number 182014). "

X26-i) Comment on ethics committee approval
"The study was approved by the Regional Ethics Committee in South-East Norway and by the Norwegian Social Science Services. All participants signed an informed consent form. "

x26-ii) Outline informed consent procedures
"At admission to the inpatient rehabilitation program, all CWP patients received a written invitation to attend an informational group meeting at which a researcher or a research assistant presented the study. Those who were interested in participating and met the inclusion criteria were given an informed consent form to sign."
The informed consent was either given to the researcher at the informational meeting or left at the reception of the rehabilitation center.
The information in the form was in line with the recommandations from the Regional Ethics Committee in South- East Norway.

X26-iii) Safety and security procedures
The safety and security procedures were evalutated and approved by the "Regional Ethics Committee in South-East Norway and by the Norwegian Social Science Services."

X27-i) State the relation of the study team towards the system being evaluated
The authors were involved in development of the intervention and the evaluation process.
